# Developing a practical neurodevelopmental prediction model for targeting high-risk very preterm infants during visit after NICU: a retrospective national longitudinal cohort study

**DOI:** 10.1186/s12916-024-03286-2

**Published:** 2024-02-16

**Authors:** Hao Wei Chung, Ju-Chieh Chen, Hsiu-Lin Chen, Fang-Yu Ko, Shinn-Ying Ho, on behalf of the Taiwan Premature Infant Follow-up Network, Jui-Hsing Chang, Kuo-Inn Tsou, Po-Nien Tsao, Shu-Chi Mu, Chyong-Hsin Hsu, Reyin Lien, Hung-Chih Lin, Chien-Chou Hsiao, Chao-Ching Huang, Chih-Cheng Chen

**Affiliations:** 1 Division of Neonatology, Department of Pediatrics, Kaohsiung Medical University Chung-Ho Memorial Hospital, Kaohsiung Medical University, Kaohsiung, Taiwan; 2 Department of Biological Science and Technology, National Yang Ming Chiao Tung University, Hsinchu, Taiwan; 3 Department of Pediatrics, Kaohsiung Municipal Siaogang Hospital, Kaohsiung Medical University, Kaohsiung, Taiwan; 4 Center for Big Data Research, Kaohsiung Medical University, Kaohsiung, Taiwan; 5 Department of Respiratory Therapy, College of Medicine, Kaohsiung Medical University, Kaohsiung, Taiwan; 6 Institute of Bioinformatics and Systems Biology, National Yang Ming Chiao Tung University, Hsinchu, Taiwan; 7 Center for Intelligent Drug Systems and Smart Bio-Devices, National Yang Ming Chiao Tung University, Hsinchu, Taiwan; 8 College of Health Sciences, Kaohsiung Medical University, Kaohsiung, Taiwan; 9 Premature Baby Foundation of Taiwan, Taipei, Taiwan

**Keywords:** Very preterm, Neurodevelopmental outcome, Prediction model, Machine learning

## Abstract

**Background:**

Follow-up visits for very preterm infants (VPI) after hospital discharge is crucial for their neurodevelopmental trajectories, but ensuring their attendance before 12 months corrected age (CA) remains a challenge. Current prediction models focus on future outcomes at discharge, but post-discharge data may enhance predictions of neurodevelopmental trajectories due to brain plasticity. Few studies in this field have utilized machine learning models to achieve this potential benefit with transparency, explainability, and transportability.

**Methods:**

We developed four prediction models for cognitive or motor function at 24 months CA separately at each follow-up visits, two for the 6-month and two for the 12-month CA visits, using hospitalized and follow-up data of VPI from the Taiwan Premature Infant Follow-up Network from 2010 to 2017. Regression models were employed at 6 months CA, defined as a decline in The Bayley Scales of Infant Development 3rd edition (BSIDIII) composite score > 1 SD between 6- and 24-month CA. The delay models were developed at 12 months CA, defined as a BSIDIII composite score < 85 at 24 months CA. We used an evolutionary-derived machine learning method (EL-NDI) to develop models and compared them to those built by lasso regression, random forest, and support vector machine.

**Results:**

One thousand two hundred forty-four VPI were in the developmental set and the two validation cohorts had 763 and 1347 VPI, respectively. EL-NDI used only 4–10 variables, while the others required 29 or more variables to achieve similar performance. For models at 6 months CA, the area under the receiver operating curve (AUC) of EL-NDI were 0.76–0.81(95% CI, 0.73–0.83) for cognitive regress with 4 variables and 0.79–0.83 (95% CI, 0.76–0.86) for motor regress with 4 variables. For models at 12 months CA, the AUC of EL-NDI were 0.75–0.78 (95% CI, 0.72–0.82) for cognitive delay with 10 variables and 0.73–0.82 (95% CI, 0.72–0.85) for motor delay with 4 variables.

**Conclusions:**

Our EL-NDI demonstrated good performance using simpler, transparent, explainable models for clinical purpose. Implementing these models for VPI during follow-up visits may facilitate more informed discussions between parents and physicians and identify high-risk infants more effectively for early intervention.

**Supplementary Information:**

The online version contains supplementary material available at 10.1186/s12916-024-03286-2.

## Background

The recent progress in perinatal and postnatal care has contributed to enhancing mortality and morbidity outcomes among preterm infants for decades. However, approximately 20% of very preterm infants (VPI) survivors still suffer from a certain degree of cognitive or motor delay at 2 years of corrected age (CA) based on the Bayley score [1]. The longitudinal follow-up program (LFUP) is regarded as the primary recommendation after neonatal intensive care unit (NICU) discharge for high-risk infants, particularly those born preterm during the first 2 years of life [2, 3]. Early detection and intervention of neurodevelopmental impairment (NDI) in high-risk infants can promote not only better outcomes but also social and economic benefits [4]. Risk factors for NDI in preterm births, such as gestational age (GA), birth body weight (BBW), bronchopulmonary dysplasia, and intraventricular hemorrhage, have been well reported [5, 6]. While evidence has substantiated the potential efficacy of innovative statistical tools for prediction models in this domain [7, 8], it is noteworthy that existing prediction models predominantly focus on aiding healthcare practitioners and families during pre-discharge counseling [9, 10].

High dropout rates exceeding 50% in LFUP make reliably evaluating VPI’s development challenging. This uncertainty and developmental status changes may make parents think their child is improving and drop out of follow-up clinics before 1 year [11]. Based on findings from the California low birthweight cohort study, early presence at a visit within the first 12 months emerged as the most significant determinant of sustained LFUP participation, alongside factors such as maternal education and proximity to the clinic [12]. Notably, lack of awareness of early intervention is significantly related to attendance [13].

The accuracy and interpretability of neurodevelopmental prediction models influence caregivers’ decision-making regarding counseling in clinics or hospitals [14, 15]. There remains a gap in prediction models for routine clinical use and NDI research [9]. The machine learning methods have produced excellent results in prediction models for a variety of diseases [16], but complex machine learning models have been the subject of recent criticisms due to their lack of transparency. Furthermore, although the performance of simpler parametric models with lots of variables is not inferior to machine learning methods, even simple algorithms like logistic regression (LR) can become complicated by including numerous predictors [17, 18]. Recently, our novel evolutionary learning method was able to establish clinical prediction models by identifying a small set of features and maximizing the prediction accuracy [19, 20].

This study aims to create practical predictive models at 6-month and 12-month visits for 24-month outcomes of CA with transparency, explainability, and transportability to help parents-physician discussion about early intervention and follow-up plans.

## Methods

### Study population

**Fig. 1:**
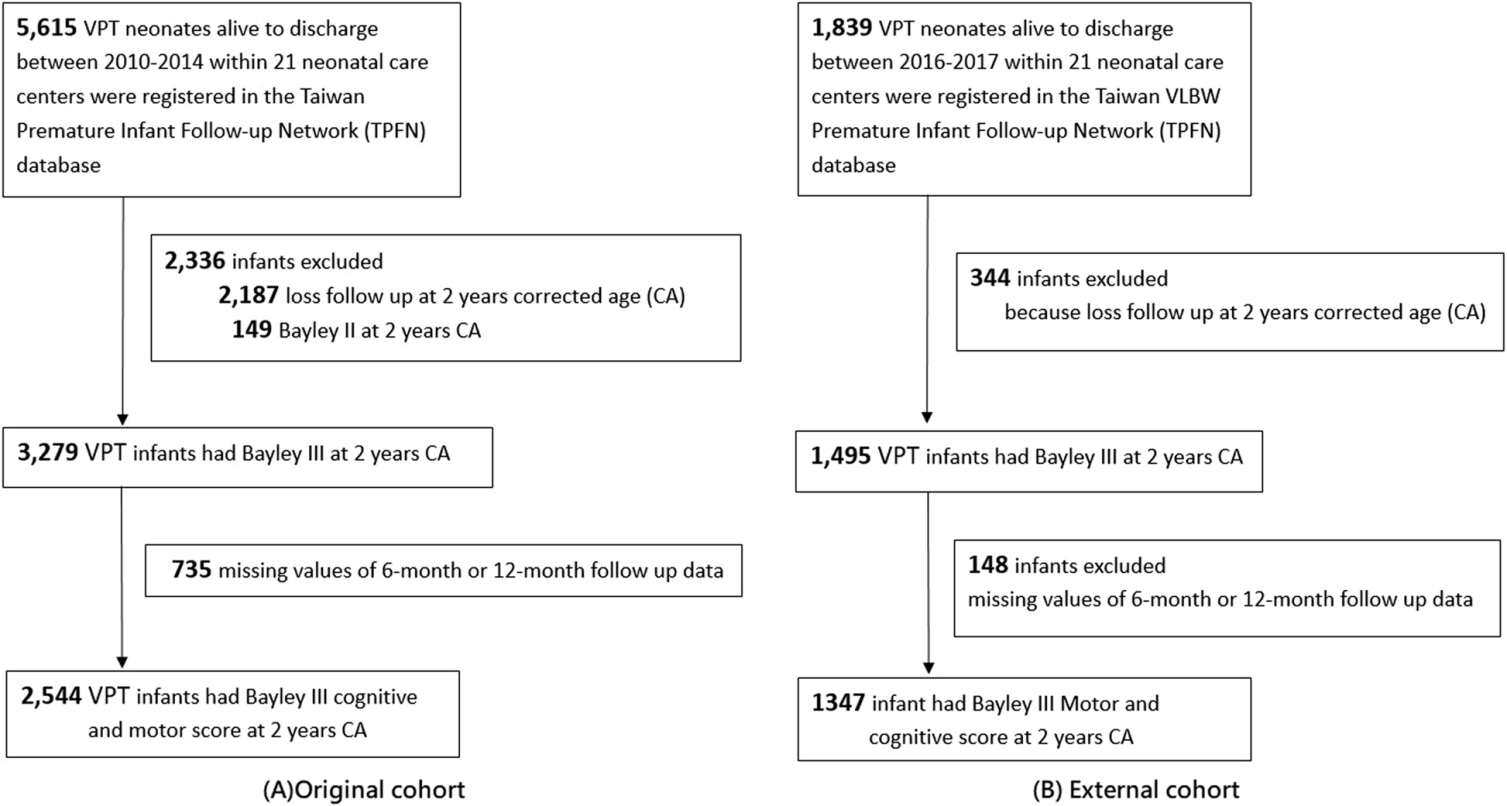
**A** Illustrated flowchart of patients’ inclusion and exclusion in the original cohort. **B** Illustrated flowchart of patients’ inclusion and exclusion in the external cohort. Bayley III, Bayley Scales of Infant Development 3rd edition; Bayley II, Bayley Scales of Infant Development 2nd edition

VPI was defined as preterm infants born before 31 weeks’ 6 days of gestation weighing 401–1500 g. Between January 1, 2010, and December 31, 2014, 5615 VPI were discharged alive from 21 neonatal care centers registered in the Taiwan Premature Infant Follow-up Network (TPFN) database, comprising the original cohort [21, 22]. For establishing accurate prediction models, we excluded 3071 infants, including infants who missed the Bayley Scales of Infant Development 3rd edition (BSIDIII) at the 6 or 12 months, as they were key variables, and those whomissed BSIDIII scores at 2 years CA alone due to main predictive outcome, or any follow-up with the Bayley Scales of Infant Development 2nd edition. There were 2544 VPI with BSIDIII cognitive and motor scores at 2 years of CA in the development cohort. An external cohort consisting of 1347 VPI was obtained from the TPFN database from January 1, 2016, to December 31,2017, using the same criteria (Fig. 1). This study was approved by the ethical standards of the Institutional Review Board of the Kaohsiung Medical University Hospital (IRB number: KMUHIRB-SV(I)- 20190008), and due to the study’s retrospective nature and the use of deidentified data, the need for written informed consent was waived.

### Neurodevelopmental outcome

Neurodevelopmental outcome in this study was based on BSIDIII score at 2 years of age of VPI. The TPFN follow-up plan included BSIDIII scores at 6, 12, and 24 months after CA by unblinded and experienced pediatric psychologists. Considering the BSIDIII score at 6 months may not be a reliable predictor of NDI at 24 months [23, 24], we designed different NDI outcome models at 6- and 12-month CA for clinical use, comprising two regression models at 6 months and two delay models at 12 months.

Two delay models were used at 12 months: the cognitive delay model (CDelay) defined NDI as a BSIDIII cognitive score of < 85 at 24 months CA and the motor delay model (MDelay) defined NDI as a BSIDIII motor score < 85 at 24 months CA. Data for both models were collected up to 12 months after CA. Two regression models were used at 6 months: the cognitive regression (CRegres) defined NDI as a BSIDIII cognitive score decline greater than one standard deviation (SD) between 6 and 24 months CA and the motor regression (MRegres) model defined NDI as a BSIDIII motor score decline greater than one SD between 6 and 24 months CA, respectively. The data used in both regression models were up to 6-month CA, and the SD of BSIDIII composite score was 15 points in two regression models.

### Prediction variables

The TPFN database contains basic demographics of parents, pregnancy, and neonatal variables at birth, hospitalization, discharge, and follow-ups at 6-, 12-, and 24-month CA. The anthropometric measures (weight, height, and head circumference) at four individual time points (admission, discharge, and 6- and 12-month CA) were redistributed into 8 intervals from less than − 3 *Z* to greater than 3 *Z*. We used the growth chart based on the INTERGROWTH-21st Project [25] at admission and discharge and the WHO Child Growth Standards [26] at the 6- and 12-month CA visit. Detailed definitions of all the variables are shown in Additional file 1.

A total of 484 variables in the TPFN database were obtained from preterm births to 12 months CA. First, we excluded variables with missing values in > 30% of the cohort, and there were 89 variables retained. We arranged missing data with imputation using the k-nearest neighbor method. However, we excluded six variables (peak bilirubin levels, blood transfusion, nasogastric tube feeding after discharge, apnea, partial pressure of oxygen, and carbon dioxide in the initial blood gas analysis) from consideration due to their absence in the external cohort dataset. Two distinct feature utilization strategies are employed in constructing prediction models. In light of the specific characteristics of random forest (RF) and lasso regression (Lasso) for handling a substantial number of predictors, an “all-features-in” approach is initially adopted for model development. Consequently, the CDelay and MDelay models are constructed with 83 variables, while the CRegres and MRegres models retain 75 variables for utilization in the Lasso and RF frameworks because the data used in regression models were only up to 6-month CA.

Second, we employ a Coarse-to-fine feature selection technique to facilitate model development, which applies to traditional machine learning methods and our novel approach. Coarse-to-fine feature selection from all recorded variables was performed as follows: Among 89 variables, the remaining 29 variables that were significantly related to NDI outcomes at 24 months based on Pearson’s and Spearman’s correlation coefficients were retained in a set of fine features for model development. The 29 variables for each prediction model and results of the correlation coefficients are shown in Additional file 2.

### Evolutionary learning method

**Fig. 2:**
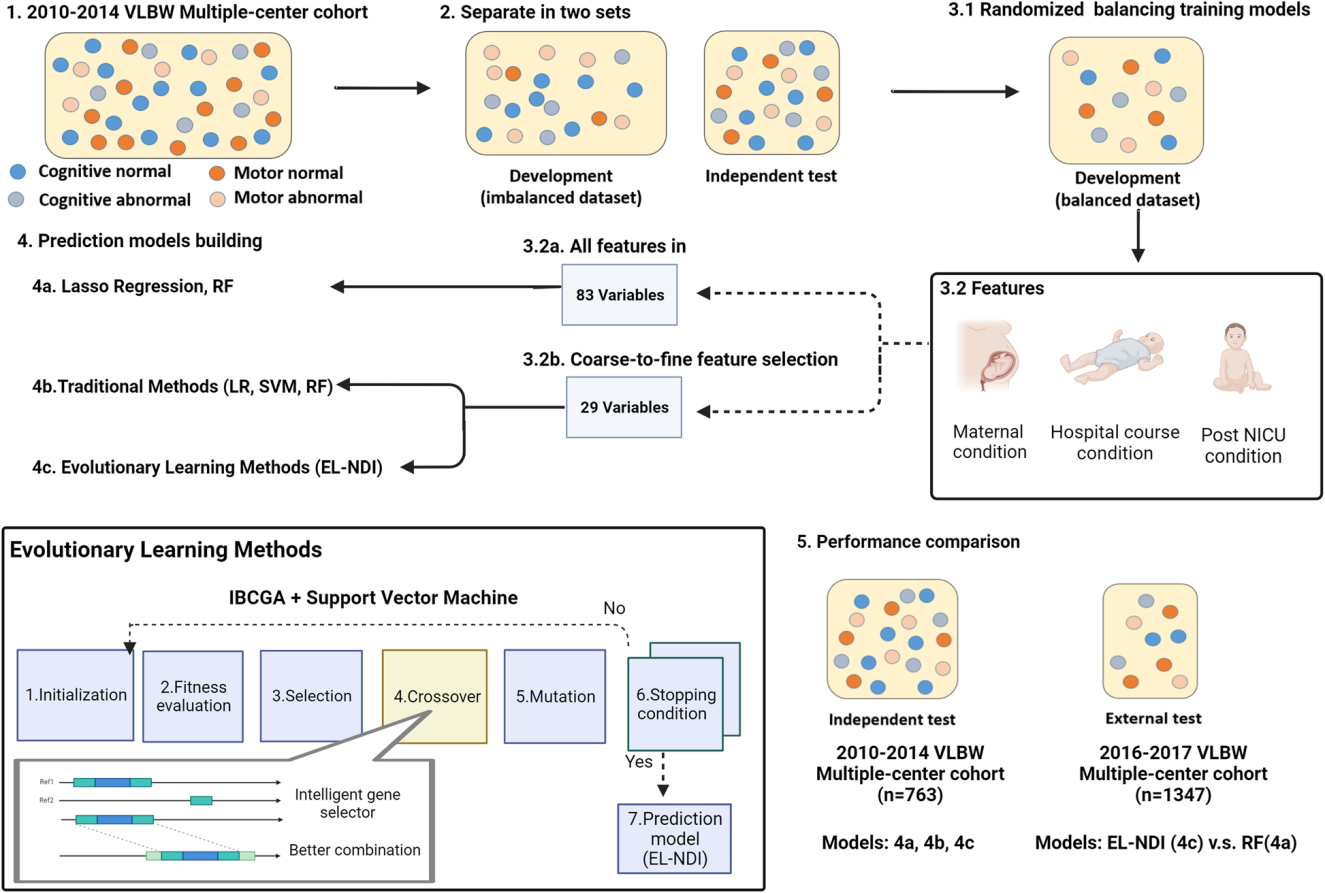
Illustrated flowchart of developing EL-NDI to predict neurodevelopmental impairment. EL-NDI utilized the inheritable bi-objective combinatorial genetic algorithm (IBCGA) alongside intelligent evolutionary algorithm (IEA) to identify feature subsets and optimize SVM parameters for maximum fitness. RF, random forest; LR, logistic regression; SVM, support vector machine

A novel evolutionary learning method, called evolutionary learning neurodevelopmental impairment (EL-NDI), was proposed to predict the NDI of VPI at 24 months of CA in this study. Figure 2 depicts the flowchart of developing EL-NDI. After excluding and including process in step 1, we divided the original cohort into development and independent test datasets at 7:3 in step 2. In the development datasets, each of the four NDI outcomes exhibited an imbalance. Subsequently, in step 3, we established four distinct balanced developmental cohorts. These cohorts were independently created by randomly pairing positive and negative cases at a 1:1 ratio drawn from the developmental dataset split in step 2. Consequently, each machine learning model was trained on a unique, balanced developmental cohort derived from the initially imbalanced development dataset, leading to variations in the cohort sizes. The 29-candidate features were obtained from the maternal, neonatal, and follow-up data with imputation using the k-nearest neighbor method. The predictive approach EL-NDI employed a widely recognized support vector machine (SVM) classifier, a statistically grounded supervised learning model. SVM are employed for classification or regression tasks through data transformation into a higher-dimensional feature space using a kernel function. The selection of both the cost parameter (*C*) and the kernel parameter (*γ*) in SVM is critical for modeling. We employed an intelligent evolutionary algorithm (IEA) [19] to determine SVM’s optimal feature selection and parameter settings. The process involved the use of the inheritable bi-objective combinatorial genetic algorithm (IBCGA) [27] in conjunction with IEA to identify a subset of features and to determine the values of the SVM parameters while maximizing the fitness function. The fitness function aimed to maximize the prediction function of the tenfold cross-validation (CV) on the training dataset. The optimal feature selection problem, denoted as *C*(*n*, *m*), entails the selection of a small subset of features (*m*) from a more extensive set of candidates (*n*), where interactions among features exist. IBCGA was employed to efficiently address this large-scale combinatorial optimization challenge to determine the value of *m*, the selected features, and the values of *C* and *γ*. For the application of IBCGA, all candidate features were encoded as binary variables for optimal feature selection. Simultaneously, the parameters (*C*, *γ*) were encoded into the chromosome for concurrent optimization. Based on the main effect difference (MED), the selected m features were ranked according to their prediction contributions. For more information about the use of IBCGA, we recommend referring to our previously published biomedicine studies [28, 29].

### Models of machine learning

We used established machine learning models to compare the EL-NDI models. The models using the R package implementation included lasso regression, logistic regression (glmnet), linear SVM (e1071), and RF (randomForest). Additionally, we combined a small set of features selected by the EL-NDI with logistic regression as the evolutionary learning logistic regression (EL-LR) model to explore the relationship between the selected features and outcomes [9]. After optimizing 19 hyperparameters for each model, we fitted the entire training set with five repetitions of tenfold cross-validation using the R caret package.

### Statistical analysis

Statistical analyses were performed using R, version 3.6.3 (R Foundation for Statistical Computing), Python, version 3.7 (Python Software Foundation), and MATLAB (version 2020a). A two-sided *p* ≤ 0.05 was considered statistically significant. We calculated 95% confidence intervals (CIs) to compare the area under the curve (AUC). Descriptive statistics were expressed as mean ± standard deviation or median (range) as appropriate. The Mann–Whitney *U* test was applied to compare continuous variables, while categorical variables were compared using Pearson *χ*^2^ analysis or Fisher’s exact test.

## Results

### Characteristics of the cohorts

The attrition rates at 24 months CA in the original and external test cohorts were 38.9% and 18.7%, respectively. According to the study design, a total of 763 VPI from 2544 VPI in original cohorts were distributed to the independent test, and there were 1347 VPI who were analyzed from external test cohort. The rest of VPI were separated into the balanced model developmental set for which the numbers were 846 and 696 VPI in the CRegres, and MRegres for 6-month CA, and 532 and 660 VPI in the CDelay, and MDelay for 12-month CA, respectively. The mean GA in the original cohort and independent and external tests were 28, 27.9, and 27.9 weeks, respectively. There was no significant difference in the NDI rates between the original and external test cohorts for all four models, with the rates being slightly higher in the external test cohort (CDelay: 16.0% vs 15.3%, *p* = 0.56; MDelay: 20.4% vs 18.5%, *p* = 0.15; CRegres: 26.1% vs 23.7%, *p* = 0.09; MRegres: 21.4% vs 19.3%, *p* = 0.12). The NDI rate, z-score distribution of BBW, GA, and sex in the original cohort, balanced development model, and independent and external tests are shown in Table 1.

**Table 1:** Gestational age, gender, birth body weight, and outcome in different models and sets

	**No. (%)**			
	2010–2014 original cohort	2010–2014 development set (balanced)	2010–2014 independent test	2016–2017 external test
Variables				
**CDelay: BSIDIII cognitive score < 85 at 24 months CA**				
	(*n* = 2544)	(*n* = 532)	(*n* = 763)	(*n* = 1347)
GA, mean (SD), weeks	28.0 (2.0)	27.8 (2.0)	27.9 (2.0)	27.9 (2.1)
Male, *N* (%)	1295 (50.9)	285 (53.6)	423 (55.4)	716 (53.2)
BBW, *N* (%)				
BBW≦ − 3Z	24 (0.9)	4 (0.7)	6 (0.8)	75 (5.6)
− 3Z < BBW ≦ − 2Z	69 (2.7)	14 (2.6)	20 (2.6)	89 (6.6)
− 2Z < BBW ≦ − Z	179 (7.1)	42 (7.9)	60 (7.9)	245 (18.2)
− Z < BBW ≦ mean	403 (15.8)	83 (15.6)	120 (15.7)	568 (42.2)
Mean < BBW ≦ Z	1161 (45.7)	237 (44.6)	342 (44.8)	349 (25.9)
Z < BBW ≦ 2Z	665 (26.1)	143 (26.9)	201 (26.4)	20 (1.4)
2Z < BBW ≦ 3Z	42 (1.6)	9 (1.7)	14 (1.8)	1 (0.1)
3Z < BBW	1 (0.1)	0 (0)	0 (0)	0 (0)
NDI outcome, *N* (%)	390 (15.3)	266 (50.0)	114 (14.9)	216 (16.0)
**MDelay: BSIDIII motor score < 85 at 24 months CA**				
	(*n* = 2544)	(*n* = 660)	(*n* = 763)	(*n* = 1347)
GA, mean (SD), weeks	28.0 (2.0)	27.7 (2.1)	27.9 (2.0)	27.9 (2.1)
Male, *N* (%)	1295 (50.9)	356 (53.9)	423 (55.4)	716 (53.2)
BBW, *N* (%)				
BBW≦ − 3Z	24 (0.9)	11 (1.7)	6 (0.8)	75 (5.6)
− 3Z < BBW ≦ − 2Z	69 (2.7)	19 (2.9)	20 (2.6)	89 (6.6)
− 2Z < BBW ≦ − Z	179 (7.1)	44 (6.6)	60 (7.9)	245 (18.2)
− Z < BBW ≦ mean	403 (15.8)	102 (15.5)	120 (15.7)	568 (42.2)
Mean < BBW ≦ Z	1161 (45.7)	296 (44.9)	342 (44.8)	349 (25.9)
Z < BBW ≦ 2Z	665 (26.1)	180 (27.3)	201 (26.4)	20 (1.4)
2Z < BBW ≦ 3Z	42 (1.6)	8 (1.2)	14 (1.8)	1 (0.1)
3Z < BBW	1 (0.1)	0 (0)	0 (0)	0 (0)
NDI outcome, *N* (%)	471 (18.5)	330 (50.0)	141 (18.5)	275 (20.4)
**CRegres: BSIDIII cognitive score declines ≧ 15 between 6 and 24 months CA**				
	(*n* = 2544)	(*n* = 846)	(*n* = 763)	(*n* = 1347)
GA, mean (SD), weeks	28.0 (2.0)	28.0 (2.0)	27.9 (2.0)	27.9 (2.1)
Male, *N* (%)	1295 (50.9)	421 (49.8)	423 (55.4)	716 (53.2)
BBW, *N* (%)				
BBW ≦ − 3Z	24 (0.9)	9 (1.0)	6 (0.8)	75 (5.6)
− 3Z < BBW ≦ − 2Z	69 (2.7)	25 (3.0)	20 (2.6)	89 (6.6)
− 2Z < BBW ≦ − Z	179 (7.1)	67 (7.9)	60 (7.9)	245 (18.2)
− Z < BBW ≦ mean	403 (15.8)	218 (25.8)	120 (15.7)	568 (42.2)
Mean < BBW ≦ Z	1161 (45.7)	370 (43.7)	342 (44.8)	349 (25.9)
Z < BBW ≦ 2Z	665 (26.1)	141 (16.7)	201 (26.4)	20 (1.4)
2Z < BBW ≦ 3Z	42 (1.6)	15 (1.8)	14 (1.8)	1 (0.1)
3Z < BBW	1 (0.1)	1 (0.1)	0 (0)	0 (0)
NDI outcome, *N* (%)	604 (23.7)	423 (50.0)	181 (23.7)	351 (26.1)
**MRegres: BSIDIII motor score declines ≧ 15 between 6 and 24 months CA**				
	(*n* = 2544)	(*n* = 696)	(*n* = 763)	(*n* = 1347)
GA, mean (SD), weeks	28.0 (2.0)	28.1 (1.9)	27.9 (2.0)	27.9 (2.1)
Male, *N* (%)	1295 (50.9)	352 (50.6)	423 (55.4)	716 (53.2)
BBW, *N* (%)				
BBW≦ − 3Z	24 (0.9)	4 (0.6)	6 (0.8)	75 (5.6)
− 3Z < BBW ≦ − 2Z	69 (2.7)	17 (2.4)	20 (2.6)	89 (6.6)
− 2Z < BBW ≦ − Z	179 (7.1)	32 (4.6)	60 (7.9)	245 (18.2)
− Z < BBW ≦ mean	403 (15.8)	100 (14.4)	120 (15.7)	568 (42.2)
Mean < BBW ≦ Z	1161 (45.7)	338 (48.6)	342 (44.8)	349 (25.9)
Z < BBW ≦ 2Z	665 (26.1)	195 (28.0)	201 (26.4)	20 (1.4)
2Z < BBW ≦ 3Z	42 (1.6)	9 (1.3)	14 (1.8)	1 (0.1)
3Z < BBW	1 (0.1)	1 (0.1)	0 (0)	0 (0)
NDI outcome, *N* (%)	491 (19.3)	342 (49.1)	149 (19.5)	288 (21.4)

### The performance of evolutionary learning and other methods in original cohort

The results of different models in validation and independent test were in Table 2.

**Table 2:** Performance of different methods in original cohort

Balanced dataset (10-CV)				Independent test	
Method	Variables numbers	ACC (%)	AUC (95% CIs)	ACC (%)	AUC (95% CIs)
**CDelay: BSIDIII cognitive score < 85 at 24 months CA**					
**All-feature-in**					
RF	83	66.0	0.68 (0.62–0.74)	76.7	0.71 (0.65–0.76)
Lasso	72	61.1	0.64 (0.59–0.69)	65.1	0.69 (0.63–0.74)
**Coarse-to-fine features selection**					
LR	29	65.6	0.70 (0.66–0.74)	69.5	0.72 (0.68–0.76)
RF	29	61.1	0.66 (0.62–0.70)	73.0	0.67 (0.63–0.71)
SVM	29	66.0	0.70 (0.64–0.74)	66.9	0.72 (0.67–0.77)
EL-NDI	10	71.9	0.74 (0.71–0.77)	77.6	0.75 (0.72–0.78)
EL-LR	10	67.1	0.72 (0.66–0.78)	70.7	0.75 (0.70–0.80)
**MDelay: BSIDIII motor score < 85 at 24 months CA**					
**All-feature-in**					
RF	83	72.7	0.76 (0.70–0.82)	73.0	0.71 (0.66–0.77)
Lasso	77	66.7	0.73 (0.65–0.81)	64.6	0.69 (0.64–0.74)
**Coarse-to-fine features selection**					
LR	29	70.3	0.77 (0.71–0.83)	69.5	0.71 (0.67–0.75)
RF	29	70.5	0.74 (0.69–0.79)	71.6	0.69 (0.66–0.72)
SVM	29	70.9	0.76 (0.71–0.81)	75.2	0.70 (0.66–0.74)
EL-NDI	4	72.4	0.78 (0.74–0.82)	75.4	0.73 (0.70–0.76)
EL-LR	4	71.7	0.77 (0.72–0.83)	72.2	0.73 (0.68–0.78)
**CRegres: BSIDIII cognitive score declines≧ 15 between 6 and 24 months CA**					
**All-feature-in**					
RF	75	71.8	0.78 (0.73–0.82)	71.7	0.78 (0.74–0.82)
Lasso	67	71.2	0.79 (0.75–0.83)	70.1	0.79 (0.76–0.83)
**Coarse-to-fine features selection**					
LR	29	72.9	0.78 (0.75–0.81)	71.6	0.80 (0.77–0.83)
RF	29	70.1	0.75 (0.72–0.78)	70.6	0.74 (0.71–0.77)
SVM	29	70.6	0.78 (0.74–0.82)	69.1	0.77 (0.73–0.80)
EL-NDI	4	72.0	0.79 (0.76–0.82)	73.0	0.81 (0.77–0.85)
EL-LR	4	71.0	0.78 (0.75–0.81)	70.8	0.80 (0.77–0.83)
**MRegres: BSIDIII motor score declines ≧ 15 between 6 and 24 months CA**					
**All-feature-in**					
RF	75	75.4	0.79 (0.76–0.82)	81.8	0.86 (0.83–0.90)
Lasso	64	71.0	0.79 (0.76–0.82)	73.3	0.82 (0.78–0.86)
**Coarse-to-fine features selection**					
LR	29	70.1	0.78 (0.74–0.82)	73.0	0.81 (0.77–0.85)
RF	29	69.7	0.76 (0.73–0.79)	69.9	0.76 (0.73–0.79)
SVM	29	71.6	0.79 (0.76–0.82)	74.1	0.81 (0.78–0.84)
EL-NDI	4	74.3	0.82 (0.79–0.85)	76.9	0.83 (0.80–0.86)
EL-LR	4	74.0	0.81 (0.78–0.84)	74.3	0.83 (0.79–0.87)

The AUC of RF with all-features-in methods in the independent test at 24 months CA was 0.71 in CDelay (sensitivity:48.0%; specificity:82.5%), 0.71 in MDelay (sensitivity:56.5%; specificity:77.3%), 0.78 in CRegres (sensitivity:72.3%; specificity: 72.6%), and 0.86 in MRegres (sensitivity:73.0%; specificity:84.5%). The AUC of EL-NDI in the independent test at 24 months CA was 0.75 in CDelay (sensitivity:50.0%; specificity:82.5%), 0.73 in MDelay (sensitivity:56.7%; specificity:79.6%), 0.81 in CRegres (sensitivity:74.6%; specificity:72.6%), and 0.83 in MRegres (sensitivity:76.5%; specificity:77.0%). EL-NDI had the highest AUC in CDelay, MDelay, and CRegres in the independent test, but RF had the highest AUC in MRegres with 75 variables (Table 2). The CDelay encompassed 10 variables: motor BSIDIII score at 12 months, cognitive BSIDIII scores at 12 months, abdominal surgery, intermittent positive pressure ventilation (IPPV) days, pH in first-time blood gas, oxygenation supply≧ 40%, head circumstance (HC) at 6 months CA, maternal education ≦ 12 years, body length (BL) at 6 months CA, and hemodynamic significant PDA. The MDelay encompassed 4 variables: motor BSIDIII scores at 12 months, cognitive BSIDIII scores at 12 months, NICU days, and post-menstrual age (PMA) while discharge. The CRegres encompassed 4 variables: cognitive BSIDIII scores at 6 months, motor BSIDIII scores at 6 months, maternal MgSO4 use, and parental education ≦ at 12 years. The MRegres encompassed 4 variables: motor BSIDIII scores at 6 months, antenatal steroid use, HC at admission, and prolonged rupture of membranes. The formulas of EL-LR to help interpret the influence of each selected variable on the predicted outcome are listed in Table 3.

**Table 3:** EL-LR formula based on variables selected by the EL-NDI

**CDelay: BSIDIII cognitive score < 85 at 24 months CA**	
Variables (*n* = 10)	
$$In\left(\frac{p}{1-p}\right)=4.39-2.22{\text{a}}-3.71{\text{b}}+1.36{\text{c}}-2.56{\text{d}}-1.14{\text{e}}+0.28{\text{f}}-1.48{\text{g}}+0.34{\text{h}}+0.8{\text{i}}-0.15{\text{j}}$$	
**a.** Motor BSIDIII score at 12 months **b**. Cognitive BSIDIII scores at 12 months **c.** Abdominal surgery **d.** IPPV days **e.** pH in 1st time blood gas **f.** Oxygenation supply≧ 40% **g.** Head circumstance at 6 months CA **h.** Maternal education ≦ 12 years **i.** Body length at 6 months CA **j.** Hemodynamic significant PDA	
**MDelay: BSIDIII motor score < 85 at 24 months CA**	
Variables (*n* = 4)	
$$In\left(\frac{p}{1-p}\right)=4.98-8.09{\text{a}}-3.31{\text{b}}+2.89{\text{c}}-1.66{\text{d}}$$	
**a.** Motor BSIDIII scores at 12 months **b.** Cognitive BSIDIII scores at 12 months **c.** NICU days **d.** PMA while discharge	
**CRegres: BSIDIII cognitive score declines ≧ 15 between 6 and 24 months CA**	
Variables (*n* = 4)	
$$In\left(\frac{p}{1-p}\right)=-4.42+8.47{\text{a}}-1.04{\text{b}}-0.65{\text{c}}+0.23{\text{d}}$$	
**a.** Cognitive BSIDIII scores at 6 months **b.** Motor BSIDIII scores at 6 months **c.** Maternal MgSO4 use **d**. Parental education ≦ 12 years	
**MRegres: BSIDIII motor score declines ≧ 15 between 6 and 24 months CA**	
Variables (*n* = 4)	
$$In\left(\frac{p}{1-p}\right)=-5.84+9.91{\text{a}}+0.52{\text{b}}+0.08{\text{c}}+0.007{\text{d}}$$	
**a.** Motor BSIDIII scores at 6 months **b**. Antenatal steroid use **c.** Head circumference at admission **d.** Prolonged rupture of membranes	
CA 6-month height/head circumstance categorized in 8 parts based on WHO boy girl growth chart *Z* score (− 3Z, − 2Z, − Z, mean, Z, 2Z, 3Z); head circumstance at admission categorized in 8 parts based on INTERGROWTH-21st very preterm size at birth reference charts *Z* score (− 3Z, − 2Z, − Z, Mean, Z, 2Z, 3Z) Note: $p$ is the probability of outcomes	

### External test validation

Among two different feature selection strategies, RF had the highest AUC in the all-features method, and EL-NDI had the highest AUC in the coarse-to-fine selection method. The AUC of RF with all-features-in methods in the external cohort at 24 months CA was 0.78 in CDelay (sensitivity:64.5%; specificity:74.8%), 0.82 in MDelay (sensitivity:71.6%; specificity:76.8%), 0.68 in CRegres (sensitivity:67.9%; specificity:59.4%), and 0.76 in MRegres (sensitivity:68.4%; specificity:77.5%). The AUC of EL-NDI in the external cohort at 24 months CA was 0.78 in CDelay (sensitivity:62.0%; specificity:77.5%), 0.82 in MDelay (sensitivity:64.7%; specificity:82.3%), 0.76 in CRegres (sensitivity:68.9%; specificity:69.9%), and 0.79 in MRegres (sensitivity:76.0%; specificity:71.2%). The performance metrics of EL-NDI and RF with all-features-in methods in external validation cohorts are shown in Table 4. The performance metrics of RF and EL-NDI in independent test and external cohort are shown in Additional file 3. Additionally, the rankings of the top five variables for four prediction models, determined independently by RF with all-features-in methods and EL-NDI, are presented in Additional file 4.

**Table 4:** Performance of EL-NDI and RF in external validation cohort (*n* = 1347)

**BSIDIII cognitive score < 85 at 24 months CA**				
	**EL-NDI** **Coarse-to-fine features selection**		**RF** **All-features-in**	
	Cognitive (**CDelay**)	Motor (**MDelay**)	Cognitive (**CDelay**)	Motor (**MDelay**)
Variables	10	4	83	83
Accuracy	75.1%	79.1%	73.2%	75.7%
AUC	0.78	0.82	0.78	0.82
95% CIs	0.75–0.82	0.79–0.85	0.73–0.80	0.79–0.85
MCC	0.321	0.425	0.310	0.415
Sensitivity	62.0%	64.7%	64.5%	71.6%
Specificity	77.5%	82.3%	74.8%	76.8%
PPV	34.5%	48.4%	32.6%	44.2%
NPV	91.4%	90.1%	91.8%	91.3%
LR +	2.75	3.65	2.56	3.08
LR −	0.47	0.48	0.47	0.37
**BSIDIII motor score declines≧ 15 between 6 and 24 months CA**				
	Cognitive (**CRegres**)	Motor (**MRegres**)	Cognitive (**CRegres**)	Motor (**MRegres**)
	4	4	75	75
Accuracy	69.6%	72.2%	61.6%	75.6%
AUC	0.76	0.79	0.68	0.76
95% CIs	0.74–0.78	0.78–0.80	0.64–0.71	0.73–0.79
MCC	0.348	0.397	0.240	0.403
Sensitivity	68.9%	76.0%	67.9%	68.4%
Specificity	69.9%	71.2%	59.4%	77.5%
PPV	44.6%	41.8%	36.9%	45.3%
NPV	86.5%	91.6%	84.1%	90.0%
LR +	2.29	2.64	1.67	3.04
LR −	0.44	0.40	0.54	0.41

## Discussion

Previous NDI prediction models had limited sample sizes and often used black box models without clearly explaining model performance [9, 10, 30]. In this large national sample of VPI, EL-NDI models utilized fewer predictors (4 and 10) with similar AUC compared with RF and lasso with All-features-in methods, specifically in external validation cohort. The neurodevelopment prediction models estimated and compared in this study were developed at the visit level, which might allow the physician to identify which individuals are at risk as well as worsen in the future.

It is difficult to compare the NDI performance between different studies because of the variety of NDI definitions and target groups [9, 10]. External validation of the Neonatal Research Network (NRN) using the estimation of five risk factors (GA at birth, exposure versus no exposure to antenatal corticosteroids, singleton versus multiple gestation, gender, and birth weight)—one of the most widely-used risk models—showed AUCs were 0.64 and 0.71 for death and severe NDI [31]. Ambalavanan et al. used 21 variables to reach AUCs of 0.66 and 0.75 for mental and psychomotor development index, respectively, at 12 to 18 months of age and showed that neural network was not superior to the logic regression model [32]. In a recent study conducted by Li et al., a machine learning prediction model based on perinatal factors, specifically utilizing SVM methodology, was found to outperform other modeling techniques such as multivariate LR, RF, and neural network analysis [7]. Notably, the SVM model in Li et al.’s study achieved an AUC of 0.7 during an independent test involving 78 very preterm infants (VPI) for composite NDI outcome, including moderate to severe cerebral palsy, cognitive or motor scores below 2 standard deviations from the norm, bilateral hearing impairment necessitating hearing aids, or bilateral blindness [7]. Conversely, our EL-NDI model excelled during a comprehensive external validation test, encompassing small sets of perinatal and post-NICU data. It emphasizes more nuanced and specific NDI outcomes, demonstrating its potential to provide more precise prognostic information.

Neuroimaging findings in preterm infants can potentially serve as a predictive indicator of adverse neurodevelopmental outcomes [33]. Moeskops et al. showed that the SVM method identified the change of brain MRIs of PMA between 30 and 40 weeks and reached AUCs of 0.80 and 0.85 for cognitive and motor BSIDIII composite scores < 85 at 2–3 years CA, respectively [34]. The neural network method reported a 100% positive predictive value and a 90.6% negative predictive value for NDI at 1 year of age using advanced brain MRIs at term-equivalent age (TEA) [35]. A prognostic study for NDI based on Denver screen test II with 109 extremely preterm infants using multimodal model *combining* electroencephalography, brain structure information, early postnatal morbidities, and perinatal factors revealed high AUCs (0.91, 95% CI, 86.4–97.0%) and demonstrated the potential of the brain functional information for prediction model [8]. The issue of overfitting and small sample sizes still needs to be addressed, regardless of whether the predictive models are based on brain function, MRI, or risk factor modeling [9, 30].

Previous investigations of cognitive and motor regression models have mostly focused on grouping and risk factors [21]. Although the development of brain trajectories correlates with future functional outcomes [36], the correlation between the degree of BSID score regression and future outcome is still unknown. However, our aim was to detect dynamic changes as early as possible and provide an opportunity to discuss the best follow-up strategy. To the best of our knowledge, this is the first prediction model for Bayley score decline between two time points for VPI.

We identify the individual impacts of each risk factor from our models to avoid black box models, which would be useful in routine clinical practice [9]. Most determinants associated with the variables in the four models align closely with findings from prior research on adverse NDI outcomes, including factors such as paternal education, gastrointestinal surgery, and duration of mechanical ventilation, [7, 37, 38]. Among anthropometric measurements in the dataset, only the BL and HC at 6 months CA were used in the CDelay. Although there is small amount of evidence to suggest that poor postnatal growth after discharge is associated with NDI in preterm infants [39, 40], the literature raises concerns regarding the efficacy of a solitary anthropometric measure, such as BL or HC, as a direct predictor of NDI in children [41]. Even though previous studies have demonstrated that very low birth weight preterm infants face various complications that may hinder their ability to achieve catch-up growth and normal neurodevelopment [41], our study explored the correlation between anthropometric measurements and other risk factors in preterm infants, particularly concerning predicting neurodevelopmental outcomes. Prolonged duration of mechanical ventilation was significantly inversely associated with NDI and brain development [42]. Surprisingly, IPPV days in the CDelay model and PMA at discharge in the MDelay model were protective factors, based on the EL-LR model. A retrospective study of factors influencing the attendance of early intervention in Iran showed that length of stay (LOS) in NICU is a primary factor that affects attendance [43]. A study of very preterm in Korea found that NICU graduates who adhered to LFUP had more severe morbidities during their NICU stay and a higher PMA at discharge [44]. Therefore, the IPPV days and PMA at discharge in CDelay and MDelay models may be associated with adherence to LFUP and early intervention, which help identify and treat health problems early in NICU graduates to improve outcomes. Subsequent research should investigate the impact of these factors on parental behavior and their consequences for these children. In regression models at 6-month CA, higher BSIDIII score in both cognitive and motor models was indicated as a risk factor. The severity of the child’s NDI was directly proportional to the lower BSIDIII score at 6 months of CA. Thus, the BSIDIII scores in VPI with more severe NDI may exhibit less variability than their counterparts [21, 23].

Despite our innovative approach, EL-NDI for CDelay still requires the inclusion of more variables to maximize accuracy compared to the other three EL-NDI models, underscoring the inherent complexity of cognitive function prediction. The difference between original and external cohorts in our study, such as a higher BSID score in two checkpoints, lower PDA ligation rate, and an increase in the proportion of lower *Z* scores for anthropometric measurements in the external cohort, may result from improved survival rates, care strategy change, and higher follow-up rates among very preterm infants in different periods [45]. Predictive models are built upon historical data and aim to make forecasts based on past knowledge. Considering the advancement in the care of VPI, this result emphasizes the limitations of existing risk factor models encompassing all parameters for predicting NDI outcomes in this field. Consequently, it becomes evident that continuous model updates are imperative to adapt to the ever-evolving landscape of medical advancements [46].

Including individuals’ current status and postnatal data within our visit-based neurodevelopment predictive modeling strategy represents a notable strength of our study. Increasing the adherence rate in follow-up has always been one of the challenges in the care of high-risk newborns, particularly premature infants, to achieve early intervention. Including postnatal data in our prediction model offers substantive proof, assuring parents that our scrutiny extends beyond historical considerations. We are equally vested in monitoring their child’s present developmental trajectory and, more crucially, comprehending how these combined elements shape the child’s prospective welfare. This approach provides a comprehensive understanding of the factors influencing neurodevelopmental outcomes and serves as the cornerstone for better decision-making between physicians and families during LFUP. We are the first prediction models for NDI in VPI with machine learning methods and external validation. Our research in developing such predictive models provides the foundation for future investigations. Within the comprehensive research framework, we have gained insights into the potential of machine learning and have recognized the limitations of current risk factor-based models in this field. Unlike conventional approaches, the EL-NDI method incorporates a model-specific signature, thereby preserving predictive accuracy across distinct cohort periods by effectively mitigating the influence of insignificant features. The integration of AI-based tools into clinical practice remains a paramount concern. AI products in clinical settings, especially those related to medical imaging, are significantly influenced by data quality [47]. We utilize routinely collected data and leverage the models to minimize parameters with reporting performance metrics, thus achieving the highest level of accuracy, which might promote ease of use and consistency in utilization. Research has shown that even before fully certifying the model’s functionalities, a convenient and effective tool can change the workflow of clinical professionals and ultimately optimize patient outcomes [48].

## Limitations

First, we carefully select predictors with strong linear correlations to optimize model efficiency, emphasizing the importance of interpretability in model development [29]. However, this coarse-to-fine feature selection might need to include the essential features that have nonlinear relationships with the outcomes and interfere with enhancing the accuracy of the model [9]. Furthermore, the available data does not include information on nonmedical practices in NICU and the child’s early environment, which are known to promote brain growth and neurodevelopment [49, 50]. Therefore, future studies will need to investigate these factors in conjunction with machine learning methods for NDI outcomes. Second, all models picked up the BSIDIII score as variables in prediction models for best performance. These models would be limited during follow-up because of a lack of trained BSID evaluators and limited budgets [51]. In our investigation, we encountered a constraint in the dataset of infant cerebral information, which solely consisted of brain sonography results from the TPFN dataset. This limitation could potentially impact the predictive efficacy of our models, as the inclusion of both morphological and functional brain data has become a recognized practice in neurocritical care for premature infants [52]. Research has demonstrated that integrating MRI or electroencephalography data with known risk factors can substantially enhance the predictive precision concerning NDI outcomes in preterm infants [8, 35]. Further prediction model studies in this field should focus on these data with large cohort validation. Fourth, despite conducting external validation, it is essential to note that the primary composition of Taiwan’s national population is East Asian. We could not test the capacity of EL-NDI on other populations. In the future, we will seek opportunities to investigate the transportability of EL-NDI.

## Conclusions

Our study demonstrated good performance of evolutionary learning models with fewer variables for cognitive and motor neurodevelopmental models at 6- and 12-month CA, respectively, in predicting NDI outcomes at 24 months CA. With a qualified assessment under an evaluation framework, our models would be helpful for targeted surveillance and optimal management of clinical progress in VPI and their families, promoting better decision-making. Further research is needed to explore the impact of these models on the attendance of LFUP in very preterm infants.

### Supplementary Information

**Additional file 1.** Variable definition. * NEC was diagnosed based on modified Bell’s stage. * IVH grade was defined based on Papile criteria.

**Additional file 2.** Utilizing Coarse-to-Fine Feature Selection with 29 Variables in Each Model Development.

**Additional file 3.** Performance of EL-NDI and RF in independent test and external cohort.

**Additional file 4.** Top five ranking variables chosen between RF with all features in methods and EL-NDI. Note: The main effect reveals the individual effect of a factor in prediction models. In calculating the influence of factors on the outcome, EL-NDI automatically computes the main effect values when the factor maximizes and minimizes its impact on the result, defining the absolute difference between these two values as MED. The most effective factor has the largest MED.

## Data Availability

The data utilized in this study were obtained through a formal application process by the Premature Foundation of Taiwan after receiving approval from KMUH IRB. The Premature Foundation of Taiwan administers the TPFN dataset. It is important to note that the data analyzed in this study cannot be publicly disclosed due to privacy regulations stated by TPFN. Access to these de-identified data is only available to TPFN members through a formal application procedure.

## Abbreviations

ACC: Accuracy
AUC: Area under curve
BE: Base deficiency
BSIDIII: Bayley Scales of Infant Development 3rd edition
BBW: Birth body weight
BL: Body length
BW: Body weight
BPD: Bronchopulmonary dysplasia
CPR: Cardiopulmonary resuscitation
CDelay: Cognitive delay model
CRegres: Cognitive regression model
CIs: Confidence intervals
CA: Corrected age
CV: Cross-validation
EL-LR: Evolutionary learning logistic regression
EL-NDI: Evolutionary learning neurodevelopmental impairment
GA: Gestational age
HC: Head circumstances
hsPDA: Hemodynamic significant patent ductus arteriosus
HFNC: High flow nasal cannula
iNO: Inhaled nitric oxide
IBCGA: Inheritable bi-objective combinatorial genetic algorithm
IRB: Institutional Review Board
IPPV: Intermittent positive pressure ventilation
IVH: Intraventricular hemorrhage
IVF: In vitro fertilization
Lasso: Lasso regression
LOS: Length of stay
LR: Likelihood ratio
LR: Logic regression
LFUP: Longitudinal follow-up program
MRI: Magnetic resonance imaging
MED: Main effect difference
MCC: Matthew’s correlation coefficient
m: Months
MDelay: Motor delay model
MRegres: Motor regression model
NCPAP: Nasal continuous positive airway pressure
NEC: Necrotizing enterocolitis
NPV: Negative predictive value
NICU: Neonatal intensive care unit
NRN: Neonatal Research Network
NDI: Neurodevelopmental impairment
PDA: Patent ductus arteriosus
PPHN: Persistent pulmonary hypertension of the newborn
PPV: Positive predictive value
PMA: Post-menstrual age
PROM: Prolonged rupture of membranes
RF: Random forest
ROP: Retinopathy of prematurity
SGA: Small for gestational age
SD: Standard deviation
SVM: Support vector machine
TPFN: Taiwan Premature Infant Follow-up Network
TEA: Term-equivalent age
VPI: Very preterm infants
WHO: World Health Organization
Z: *Z* Score
