# Supplementary material for: Developing a practical neurodevelopmental prediction model for targeting high-risk very preterm infants during visit after NICU: a retrospective national longitudinal cohort study
Source: BMC Med. 2024 Feb 16;22:68. doi: 10.1186/s12916-024-03286-2 (PMC10870669; doi:10.1186/s12916-024-03286-2)
Supplement: Supplementary file 1 — Additional file 1. Variable definition. * NEC was diagnosed based on modified Bell’s stage. * IVH grade was defined based on Papile criteria. [file 12916_2024_3286_MOESM1_ESM.docx]

**Additional files 1. Variable definition**

| Predictor variables | Definition |
| --- | --- |
| **Parents variables for time point of birth** | |
| Marriage | Binary; Marriage or not |
| Maternal Nation | Binary; Natural Mandarin speaker or not |
| Maternal age | Maternal age in years |
| Paternal age | Paternal age in years |
| Maternal education | Binary; Education ≦12 years or not |
| Paternal education | Binary; Education ≦12 years or not |
| **Pregnant variables for time point before delivery** | |
| Gestational age | Gestational age in completed weeks |
| First child | Binary; First child or not |
| Single gestation | Binary; Single or not |
| In vitro fertilization (IVF) | Binary; IVF or not |
| Preeclampsia | Binary; Preeclampsia or not, as reported on obstetric records |
| Prolonged rupture of membranes (PROM) | Binary; Rupture of membranes more than 18 hours or not, as reported on obstetric records |
| MgSO4 | Binary; MgSO4 administered to mother before delivery or not, as reported on obstetric records |
| Antenatal steroid | Class variables for doses of antenatal steroid to accelerate pulmonary maturity.  (0 = none ,1= 1 dose, 2= 2 doses or more) |
| **Neonates variables for time point of birth** | |
| Cesarean delivery | Binary; Cesarean delivery or not |
| Male gender | Binary; Male or not |
| Neonate transfer | Binary; Transfer for level 3 just after birth or not |
| Birth weight | Class variables for z score of gestational age birth chart  0 = measurement ≦ -3Z  1 = -3Z < measurement ≦ -2Z  2 = -2Z < measurement ≦ - Z  3 = -Z < measurement ≦ Mean  4 = Mean < measurement ≦ Z  5 = Z < measurement ≦ 2Z  6 = 2Z < measurement ≦ 3Z  7 = 3Z< measurement |
| Birth height | Class variables for z score of gestational age birth chart  0 = measurement ≦ -3Z  1 = -3Z < measurement ≦ -2Z  2 = -2Z < measurement ≦ - Z  3 = -Z < measurement ≦ Mean  4 = Mean < measurement ≦ Z  5 = Z < measurement ≦ 2Z  6 = 2Z < measurement ≦ 3Z  7 = 3Z< measurement |
| Birth head circumference | Class variables for z score of gestational age birth chart  0 = measurement ≦ -3Z  1 = -3Z < measurement ≦ -2Z  2 = -2Z < measurement ≦ - Z  3 = -Z < measurement ≦ Mean  4 = Mean < measurement ≦ Z  5 = Z < measurement ≦ 2Z  6 = 2Z < measurement ≦ 3Z  7 = 3Z < measurement |
| SGA | Small for gestational age (below the 10th percentile). Based on Taiwan birth cohort study |
| Birth CPR | Binary; Resuscitation was including chest compression, intubation, administration medication. |
| 1min Apgar score | Apgar score at 1min |
| 5min Apgar score | Apgar score at 5min |
| **Neonates variables for time point of admission** | |
| 1^st^ time Body temperature | First body temperature showed as degrees Celsius (°C) |
| 1^st^ time Blood Sugar | First time blood sugar measured as mg/dL |
| pH in 1st time blood gas | First blood pH levels no matter from vein or artery |
| PO2 in 1st time blood gas | First blood oxygen levels no matter from vein or artery showed as mmHg |
| PCO2 in 1st time blood gas | First blood carbon dioxygen levels no matter from vein or artery showed as mmHg |
| HCO3 in 1st time blood gas | First sodium bicarbonate levels no matter from vein or artery showed as mmol/L |
| BE in 1st time blood gas | First base deficiency levels no matter from vein or artery |
| **Medication and treatment variables during the hospitalization** | |
| Surfactant | Binary; Administration of surfactant to infant or not |
| Indomethacin | Binary; Administration of Indomethacin to infant or not |
| iNO | Binary; Administration of inhalation NO to infant or not |
| Ibuprofen | Binary; Administration of Ibuprofen to treat PDA or not |
| Aminophylline | Binary; Administration of Aminophylline to treat apnea or not |
| Blood transfusion | Binary; Received any episode of blood transfusion |
| Postnatal steroid | Binary; Administration of any type of steroid to treat BPD or not |
| ROP treatment | Binary; Any treatment including laser therapy or cryotherapy for ROP or not |
| **Lab data variables during the hospitalization** | |
| Peak bilirubin level | The highest bilirubin level showed as mg/dl |
| **Diagnosis variables during the hospitalization** | |
| Perinatal infection | Binary; Perinatal infection or not |
| Sepsis | Binary; Any episode of bacteria culture proven sepsis or not |
| hsPDA | Binary; Hemodynamic significant PDA was diagnosed or not |
| Apnea of prematurity | Binary; Apnea of prematurity was recorded with intervention no matter medication or ventilation |
| ROP Stage 3 | Binary; Any Stage more severe than stage 3 of retinopathy of prematurity without Plus disease |
| ROP Plus disease | Binary; Any stage of retinopathy of prematurity with Plus disease |
| Gastrointestinal perforation | Binary; Any episode of gastrointestinal perforation or not |
| *NEC | Class variables for the severity of NEC,  0= none or stage I, 1 = stage II, 2= more than stage III |
| BPD | Binary; BPD was defined according to the National Institute of Child Health and Human Development criteria |
| Air leak syndrome | Binary; Any episode of pneumothorax, pneumomediastinum, pneumopericardium, pneumoperitoneum or not |
| PPHN | Binary; Persistent pulmonary hypertension of the newborn was diagnosed or not |
| **Operation variables during the hospitalization** | |
| PDA ligation | Binary; Received PDA ligation or not |
| Cardiac surgery | Binary; Received cardiac surgery except PDA ligation or not |
| Abdominal surgery | Binary; Received abdominal surgery or not |
| External ventricular drain | Binary; Received external ventricular drain or not |
| **Respiratory variables during the hospitalization** | |
| High oxygenation supply | Binary; Peak oxygenation over 40% or not |
| IPPV day | Days on intubation during hospitalization |
| Respiratory support days | Days on oxygen or HFNC or NCPAP or ventilator |
| PMA without respiratory support | Age presents as weeks on post maternal age without oxygen or HFNC or NCPAP or any ventilator. |
| **Brain sonography variables during hospitalization** | |
| Intraventricular hemorrhage* | Class variables for the severity,  0= no hemorrhage, 1= intraventricular hemorrhage grades I and II, 2= intraventricular hemorrhage grades III and IV |
| Post hemorrhage hydrocephalus | Binary; Post hemorrhage hydrocephalus was diagnosed during hospitalization |
| Ventriculomegaly | Binary; Ventriculomegaly was diagnosed |
| Cystic periventricular leukomalacia | Binary; Any cystic periventricular leukomalacia was diagnosed |
| Periventricular echogenicity | Binary; Any episode of Periventricular echogenicity was diagnosed |
| Choroid plexus hemorrhage | Binary; Any episode of Choroid plexus hemorrhage was diagnosed |
| **Discharge variables** | |
| Discharge weight | Class variables for z score of very preterm growth chart  0 = measurement ≦ -3Z  1 = -3Z < measurement ≦ -2Z  2 = -2Z < measurement ≦ - Z  3 = -Z < measurement ≦ Mean  4 = Mean < measurement ≦ Z  5 = Z < measurement≦ 2Z  6 = 2Z < measurement≦ 3Z  7 = 3Z< measurement |
| Discharge height | Class variables for z score of very preterm growth chart  0 = measurement ≦ -3Z  1 = -3Z < measurement ≦ -2Z  2 = -2Z < measurement ≦ - Z  3 = -Z < measurement ≦ Mean  4 = Mean < measurement ≦ Z  5 = Z < measurement≦ 2Z  6 = 2Z < measurement≦ 3Z  7 = 3Z< measurement |
| Discharge head circumference | Class variables for z score of very preterm growth chart  0 = measurement ≦ -3Z  1 = -3Z < measurement ≦ -2Z  2 = -2Z < measurement ≦ - Z  3 = -Z < measurement ≦ Mean  4 = Mean < measurement ≦ Z  5 = Z < measurement≦ 2Z  6 = 2Z < measurement≦ 3Z  7 = 3Z< measurement |
| NICU days | NICU hospitalization duration presents as days |
| Discharge post maternal age | Post maternal age at discharge point presents as weeks on post maternal age |
| O2 supply after discharge | Binary; Administer oxygen after discharge or not |
| NG feeding after discharge | Binary; Administer NG feeding after discharge or not |
| Hearing impairment | Binary; Any degree of hearing impairment was diagnosed before discharge or not |
| **Variables while regular follow up at 6 months CA** | |
| Weight | Class variables for z score of WHO Boy/Girl growth chart  0 = measurement ≦ -3Z  1 = -3Z < measurement ≦ -2Z  2 = -2Z < measurement ≦ - Z  3 = -Z < measurement ≦ Mean  4 = Mean < measurement ≦ Z  5 = Z < measurement≦ 2Z  6 = 2Z < measurement≦ 3Z  7 = 3Z< measurement |
| Height | Class variables for z score of WHO Boy/Girl growth chart  0 = measurement ≦ -3Z  1 = -3Z < measurement ≦ -2Z  2 = -2Z < measurement ≦ - Z  3 = -Z < measurement ≦ Mean  4 = Mean < measurement ≦ Z  5 = Z < measurement≦ 2Z  6 = 2Z < measurement≦ 3Z  7 = 3Z< measurement |
| Head circumference | Class variables for z score of WHO Boy/Girl growth chart  0 = measurement ≦ -3Z  1 = -3Z < measurement ≦ -2Z  2 = -2Z < measurement ≦ - Z  3 = -Z < measurement ≦ Mean  4 = Mean < measurement ≦ Z  5 = Z < measurement≦ 2Z  6 = 2Z < measurement≦ 3Z  7 = 3Z< measurement |
| Caretaker | Binary; Main caretaker is natural mother or not |
| Muscle tone | Class variables for muscle tone as medical recorded at 6 months CA  0=hypotonia, 1= normal, 2= hypertonia |
| Vision impairment | Binary; Ipsilateral or both eye blindness at 6 months CA was diagnosed by Ophthalmologist or not |
| BSID-III Cognitive score | Bayley-III Cognitive composite scores: experienced pediatric psychologists evaluated all participants at 6months CA using the Bayley Scales of Infant Development 3rd edition |
| BSID-III Motor score | Bayley-III Motor composite scores: experienced pediatric psychologists evaluated all participants at CA 6month using the Bayley Scales of Infant Development 3rd edition |
| **Variables while regular follow up at 12-months CA** | |
| Weight | Class variables for z score of WHO Boy/Girl growth chart  0 = measurement ≦ -3Z  1 = -3Z < measurement ≦ -2Z  2 = -2Z < measurement ≦ - Z  3 = -Z < measurement ≦ Mean  4 = Mean < measurement ≦ Z  5 = Z < measurement≦ 2Z  6 = 2Z < measurement≦ 3Z  7 = 3Z< measurement |
| Height | Class variables for z score of WHO Boy/Girl growth chart  0 = measurement ≦ -3Z  1 = -3Z < measurement ≦ -2Z  2 = -2Z < measurement ≦ - Z  3 = -Z < measurement ≦ Mean  4 = Mean < measurement ≦ Z  5 = Z < measurement≦ 2Z  6 = 2Z < measurement≦ 3Z  7 = 3Z< measurement |
| Head circumference | Class variables for z score of WHO Boy/Girl growth chart  0 = measurement ≦ -3Z  1 = -3Z < measurement ≦ -2Z  2 = -2Z < measurement ≦ - Z  3 = -Z < measurement ≦ Mean  4 = Mean < measurement ≦ Z  5 = Z < measurement≦ 2Z  6 = 2Z < measurement≦ 3Z  7 = 3Z< measurement |
| Vision impairment | Ipsilateral or both eye blindness at 12 months CA was diagnosed by Ophthalmologist or not |
| BSID-III Cognitive score | Bayley-III Cognitive composite scores: experienced pediatric psychologists evaluated all participants at 12months CA using the Bayley Scales of Infant Development 3rd edition |
| BSID-III Motor score | Bayley-III Motor composite scores: experienced pediatric psychologists evaluated all participants at 12months CA using the Bayley Scales of Infant Development 3rd edition |
| Caretaker | Binary; Main caretaker is natural mother or not |
| Muscle tone | Muscle tone was categorized as three degrees, hypotonia, normal, hypertonia as medical recorded at 12 months  0=hypotonia, 1= normal, 2= hypertonia |
| * NEC was diagnosed based on modified Bell's stage  * IVH grade was defined based on Papile criteria | |
