# Supplementary material for: Developing a practical neurodevelopmental prediction model for targeting high-risk very preterm infants during visit after NICU: a retrospective national longitudinal cohort study
Source: BMC Med. 2024 Feb 16;22:68. doi: 10.1186/s12916-024-03286-2 (PMC10870669; doi:10.1186/s12916-024-03286-2)
Supplement: Supplementary file 2 — Additional file 2. Utilizing Coarse-to-Fine Feature Selection with 29 Variables in Each Model Development. [file 12916_2024_3286_MOESM2_ESM.docx]

**Additional file 2. Utilizing Coarse-to-Fine Feature Selection with 29 Variables in Each Model Development**

| **CDelay: BSIDIII Cognitive score < 85 at 24 months CA** | | | | | |
| --- | --- | --- | --- | --- | --- |
|  |  | No (%) |  |  |  |
| Variables | Coefficient (p value) | Original  cohort  (n=2544) | Development  (Balance)  (n=532) | Independent  Test  (n=763) | External cohort  (n=1347) |
| **Parents variables for time point of birth** | | | | | |
| Maternal education less than 12 years, n (%) | -0.145 (2.22E-16) | 810(31.8) | 183(34.4) | 252(33.0) | 533(39.6) |
| Paternal education less than 12 years, n (%) | -0.126  (1.01E-12) | 847(33.3) | 188(35.3) | 261(34.2) | 532(39.5) |
| **Pregnant variables for time point before delivery** | | | | | |
| Gestational age, mean (SD), wk | 0.121  (6.96E-12) | 28.0(2.0) | 27.8(2.0) | 27.9(2.0) | 27.9(2.1) |
| **Neonate variables for time point of admission** | | | | | |
| pH in 1st time blood gas, mean (SD) | 0.082  (3.67E-06) | 7.29(0.1) | 7.29(0.1) | 7.30(0.1) | 7.2(1.1) |
| **Medication and treatment variables during the hospitalization** | | | | | |
| Surfactant, n (%) | -0.091  (2.63E-07) | 1061(41.7) | 230(43.2) | 331(43.4) | 536(39.8) |
| Blood transfusion  (%) | -0.087  (9.41E-07) | 1917(75.4) | 318(59.8) | 589(77.2) | none |
| ROP treatment, n (%) | -0.098  (3.44E-08) | 279(10.9) | 63(11.8) | 85(11.1) | 156(11.6) |
| **Brain sonography variables during hospitalization** | | | | | |
| IVH, n (%) | -0.081  (5.15E-06) | None:  1608(63.2)  Gr I、II:  769 (30.2)  Gr III、IV:  167 (6.6) | None:  320 (60.2)  Gr I、II:  164 (30.8)  Gr III、IV:  48 (9.0) | None:  485 (63.6)  Gr I、II:  232 (30.4)  Gr III、IV:  46 (6.0) | None:  905(67.2)  Gr I、II:  370(27.5)  Gr III、IV:  72(5.3) |
| **Diagnosis variables during the hospitalization** | | | | | |
| hsPDA, n (%) | -0.077  (1.47E-05) | 1199(47.1) | 267(50.2) | 354(46.3) | 797(59.2) |
| ROP Stage.3, n (%) | -0.081  (5.28E-06) | 241(9.5) | 59(11.6) | 70(9.2) | 104(7.7) |
| ROP Plus disease  n (%) | -0.080  (7.16E-06) | 86(3.4) | 22(4.1) | 34(4.5) | 68(5) |
| BPD, n (%) | -0.079  (6.86E-06) | 1161(45.6) | 258(48.5) | 362(47.4) | 695(51.6) |
| **Operation variables during the hospitalization** | | | | | |
| Abdominal surgery | -0.071  (5.89E-05) | 96 (3.8) | 24 (4.5) | 33(4.3) | 45(3.3) |
| **Respiratory variables during the hospitalization** | | | | | |
| High oxygenation supply, n (%) | -0.097  (4.02E-08) | 1078 (42.4) | 267 (50.2) | 328(43.0) | 530(39.3) |
| IPPV days, mean (SD), d | -0.118  (2.92E-11) | 12.2(21.3) | 13.07(24.1) | 11.6(20.4) | 10.2(17.3) |
| Respiratory support days, mean (SD), d | -0.163  (0.000) | 58.4(39.3) | 61.9(40.6) | 59.8(40.3) | 60.3(36.3) |
| PMA without respiratory support, mean (SD),wk | -0.152  (0.000) | 35.8(4.3) | 36.2(4.5) | 36.1(4.6) | 36.1(3.9) |
| **Discharge variables** | | | | | |
| NICU days, mean (SD), d | -0.156  (0.000) | 54.9(37.2) | 58.1(35.2) | 55.9(35.0) | 57.7(35.6) |
| PMA while discharge, mean (SD), wk | -0.147  (0.000) | 39.0(4.1) | 39.3(4.2) | 39.2(4.2) | 39.4(4.4) |
| **Variables while regular follow up at 6 months CA** | | | | | |
| BW,  Z scores, n(%) | | | | | |
| BW≦-3Z | 0.075  (2.10E-05) | 22(0.9) | 4(0.8) | 7(0.9) | 55(4.1) |
| -3Z< BW≦-2Z |  | 115 (4.5) | 28 (5.3) | 24 (3.2) | 98(7.3) |
| -2Z< BW≦-Z |  | 216(8.5) | 48(9.0) | 71(9.3) | 259(19.2) |
| -Z< BW≦Mean |  | 484(19.1) | 114(21.4) | 162(21.2) | 386(28.7) |
| Mean< BW≦Z |  | 752(29.6) | 156(29.4) | 217(28.4) | 357(26.5) |
| Z< BW≦2Z |  | 649(25.5) | 123(23.1) | 192(25.2) | 157(11.7) |
| 2Z< BW≦3Z |  | 249(9.8) | 48(9.0) | 73(9.6) | 31(2.3) |
| 3Z< BW |  | 57(2.1) | 11(2.0) | 17(2.2) | 4(0.3) |
| BL,  Z scores, n(%) | | | | | |
| BL≦-3Z | 0.086  (1.10E-06) | 21(0.8) | 4(0.8) | 4(0.5) | 73(5.4) |
| -3Z< BL≦-2Z |  | 171(6.8) | 37(7.0) | 43(5.6) | 126(9.4) |
| -2Z< BL≦-Z |  | 244(9.6) | 55(10.3) | 95(12.4) | 260(19.3) |
| -Z< BL≦Mean |  | 504(19.8) | 108(20.3) | 156(20.5) | 351(26.1) |
| Mean< BL≦Z |  | 691(27.2) | 147(27.6) | 194(25.5) | 321(23.8) |
| Z< BL≦2Z |  | 581(22.8) | 117(22.0) | 165(21.6) | 162(12.0) |
| 2Z< BL≦3Z |  | 263(10.3) | 49(9.2) | 86(11.3) | 40(3.0) |
| 3Z< BL |  | 69(2.7) | 15(2.8) | 20(2.6) | 14(1.0) |
| HC,  Z scores, n(%) | | | | | |
| HC≦-3Z | 0.119  (2.08E-11) | 13(0.5) | 1(0.2) | 4(0.5) | 85(6.3) |
| -3Z< HC≦-2Z |  | 113(4.4) | 26(4.9) | 32(4.2) | 109(8.1) |
| -2Z< HC≦-Z |  | 237(9.4) | 59(11.1) | 80(10.5) | 289(21.5) |
| -Z< HC≦Mean |  | 482(19.0) | 113(21.2) | 142(18.6) | 386(28.7) |
| Mean< HC≦Z |  | 809(31.8) | 172(32.3) | 236(30.9) | 295(21.9) |
| Z< HC≦2Z |  | 601(23.6) | 106(19.9) | 180(23.7) | 147(10.9) |
| 2Z< HC≦3Z |  | 245(9.6) | 45(8.5) | 78(10.2) | 32(2.4) |
| 3Z< HC |  | 44(1.7) | 10(1.9) | 11(1.4) | 3(0.2) |
| BSID-III Cognitive score, mean (SD) | 0.236(0) | 95.9(12.7) | 93.3(14.35) | 95.9(12.6) | 97.8(12.6) |
| BSID-III Motor score, mean (SD) | 0.224(0) | 92.5(15.1) | 89.4(17.2) | 92.6(14.9) | 94.0(14.5) |
| **Variables while regular follow up at 12 months CA** | | | | | |
| BW,  Z scores, n(%) | | | | | |
| BW≦-3Z | 0.104  (4.34E-09) | 7(0.3) | 0(0) | 3(0.4) | 39(2.9) |
| -3Z< BW≦-2Z |  | 78(3.1) | 29(5.5) | 17(2.2) | 131(9.8) |
| -2Z< BW≦-Z |  | 204(8.0) | 45(8.5) | 61(8.0) | 299(22.4) |
| -Z< BW≦Mean |  | 560(22.0) | 130(24.4) | 176(23.1) | 363(27.2) |
| Mean< BW≦Z |  | 869(34.1) | 163(30.6) | 266(34.9) | 332(24.9) |
| Z< BW≦2Z |  | 616(24.2) | 118(22.2) | 178(23.3) | 115(8.6) |
| 2Z< BW≦3Z |  | 183(7.2) | 41(7.7) | 53(7.0) | 23(1.7) |
| 3Z< BW |  | 27(1.1) | 6(1.1) | 9(1.1) | 10(0.7) |
| BL,  Z scores, n(%) | | | | |  |
| BL≦-3Z | 0.078  (9.78E-06) | 15(0.6) | 3(0.5) | 8 (1.1) | 63(4.7) |
| -3Z< BL≦-2Z |  | 124(4.9) | 36(6.8) | 34 (4.5) | 131(9.8) |
| -2Z< BL≦-Z |  | 284(11.2) | 64(12.0) | 96 (12.6) | 299 (22.4) |
| -Z< BL≦Mean |  | 501(19.7) | 100(18.8) | 152 (19.9) | 363(27.2) |
| Mean< BL≦Z |  | 785(30.9) | 160(30.1) | 228 (29.9) | 332 (24.9) |
| Z< BL≦2Z |  | 566(22.2) | 106(19.9) | 159 (20.8) | 115(8.6) |
| 2Z< BL≦3Z |  | 211(8.3) | 47(8.8) | 67 (8.7) | 23(1.7) |
| 3Z< BL |  | 58(2.2) | 16(3.1) | 19 (2.5) | 10 (0.7) |
| BSID-III Cognitive score, mean (SD) | 0.348  (0) | 95.9(12.7) | 94.7(14.1) | 98.8(11.6) | 99.0(12.6) |
| BSID-III Motor score, mean (SD) | 0.341  (0) | 92.5(15.1) | 87.5(16.2) | 92.4(13.0) | 92.3(13.5) |
| **MDelay: BSIDIII Motor score < 85 at 24 months CA** | | | | | |
|  |  | No (%) |  | |  |
| Variables |  | Original  cohort  (n=2544) | Train  (Balance)  (n=660) | Independent  Test  (n=763) | External cohort  (n=1347) |
| **Pregnant variable for time point before delivery** | | | | |  |
| Gestational age, mean (SD), wk | 0.154  (0) | 28.0(2.0) | 27.7(2.1) | 27.9(2.0) | 27.9(2.1) |
| **Neonates’ variables for time point of admission** | | | | | |
| BW,  Z scores, n(%) | | | | | |
| BW≦-3Z | -0.046  (0.010) | 24(0.9) | 11(1.7) | 6(0.8) | 75(5.6) |
| -3Z< BW≦-2Z |  | 69(2.7) | 19(2.9) | 20(2.6) | 89(6.6) |
| -2Z< BW≦-Z |  | 179(7.0) | 44(6.7) | 60(7.9) | 245(18.2) |
| -Z< BW≦Mean |  | 403(15.8) | 102(15.5) | 120(15.7) | 568(42.2) |
| Mean< BW≦Z |  | 1161(45.6) | 296(44.8) | 342(44.8) | 349(25.9) |
| Z< BW≦2Z |  | 665(26.2) | 180(27.2) | 201(26.3) | 20(1.4) |
| 2Z< BW≦3Z |  | 42(1.7) | 8(1.2) | 14(1.9) | 1(0.1) |
| 3Z< BW |  | 1(0.1) | 0(0) | 0(0) | 0(0.0) |
| 1min Apgar score, mean (SD) | 0.076  (1.65E-05) | 5.53(2.0) | 5.41(2.1) | 5.54(2.0) | 5.6(2.0) |
| 5min Apgar score, mean (SD) | 0.081  (5.25E-06) | 7.6(1.6) | 7.5(1.6) | 7.6(1.6) | 7.8(1.7) |
| 1^st^ time Blood Sugar, mg/dl mean (SD) | -0.081  (5.43E-06) | 75.0(34.0) | 76.2(35.4) | 76.4(35.3) | 73.8(34.7) |
| pH in 1st time blood gas, mean (SD) | 0.072  (4.95E-05) | 7.3(0.1) | 7.3(0.1) | 7.3(0.1) | 7.2(1.1) |
| BE in 1st time blood gas, mean (SD), mEq/L | 0.043  (0.016) | -4.0(4.5) | -4.4(4.1) | -4.0(5.4) | 4.1(4.1) |
| **Lab data variables during the hospitalization** | | | | | |
| Peak bilirubin level, mean (SD), mg/dl | 0.042  (0.017) | 9.0(3.0) | 8.9(2.9) | 9.1(2.9) | none |
| **Medication and treatment variables during the hospitalization** | | | | | |
| ROP treatment, n (%) | -0.135  (2.00E-14) | 279(10.9) | 101(15.3) | 85(11.1) | 156(11.6) |
| **Diagnosis variables during the hospitalization** | | | | | |
| hsPDA, n (%) | -0.086  (1.27E-06) | 1199(47.1) | 322(48.8) | 365(47.8) | 797(59.2) |
| NEC, n (%) | 0.061  (0.001) | none or stage I 2373(93.3)  stage II  123 (4.8)  ≧stage III  48 (1.9) | none or stage I  613 (92.9)  stage II  28 (4.2)  ≧stage III  19 (2.9) | none or stage I  707 (92.7)  stage II  42 (5.5)  ≧stage III  14 (1.8) | none or stage I  1273(94.5)stage II  55(4.1)  ≧stage III  19(1.4) |
| BPD, n (%) | -0.144  (4.44E-16) | 1161(45.6) | 348(52.7) | 362(47.4) | 695(51.6) |
| **Operation variables during the hospitalization** | | | | | |
| PDA ligation, n (%) | -0.123  (4.00E-1) | 474(18.6) | 139(21.1) | 155(20.3) | 188(14.0) |
| **Respiratory variables during the hospitalization** | | | | | |
| IPPV days, mean (SD), d | -0.148  (0) | 12.2(21.3) | 15.4(25.4) | 11.6(20.4) | 10.2(17.3) |
| Respiratory support days, mean (SD), d | -0.219  (0) | 58.4(39.3) | 65.9(44.8) | 59.8(40.3) | 60.3(36.3) |
| PMA without respiratory support, mean (SD),wk | -0.175  (0) | 35.8(4.3) | 36.6(5.1) | 36.1(4.6) | 36.1(3.9) |
| **Discharge variables** | | | | | |
| NICU days, mean (SD), d | -0.199  (0) | 54.9(37.2) | 59.66(37.07) | 56.8(45.61) | 57.7(35.6) |
| PMA while discharge, mean (SD), wks | -0.175  (0) | 38.9(4.0) | 39.6(4.9) | 39.0(4.2) | 39.4(4.4) |
| HC  Z scores, n, (%) | | | | | |
| HC≦-3Z | 0.044  (0.013) | 177(7.0) | 54(8.2) | 59(7.7) | 221(16.5) |
| -3Z< HC≦-2Z |  | 297(11.7) | 88(13.3) | 82(10.8) | 239(17.8) |
| -2Z< HC≦-Z |  | 397(15.6) | 109(16.5) | 134(17.6) | 334(24.9) |
| -Z< HC≦Mean |  | 810(31.8) | 188(28.5) | 236(30.9) | 340(25.4) |
| Mean< HC≦Z |  | 651(25.6) | 160(24.2) | 179(23.5) | 157(11.7) |
| Z< HC≦2Z |  | 175(6.9) | 54(8.2) | 56(7.3) | 39(2.9) |
| 2Z< HC≦3Z |  | 31(1.2) | 6(0.9) | 13(1.7) | 7(0.5) |
| 3Z< HC |  | 6(0.2) | 1(0.2) | 4(0.5) | 2(0.1) |
| **Variables while regular follow up at 6 months CA** | | | | | |
| BW,  Z scores, n (%) | | | | | |
| BW≦-3Z | 0.080  (7.09E-06) | 22(0.9) | 7(1.1) | 7(0.9) | 55(4.1) |
| -3Z< BW≦-2Z |  | 115(4.5) | 45(6.8) | 24(3.2) | 98(7.3) |
| -2Z< BW≦-Z |  | 216(8.5) | 63(9.6) | 71(9.3) | 259(19.2) |
| -Z< BW≦Mean |  | 484(19.1) | 129(19.5) | 162(21.2) | 386(28.7) |
| Mean< BW≦Z |  | 752(29.6) | 186(28.2) | 217(28.4) | 357(26.5) |
| Z< BW≦2Z |  | 649(25.5) | 155(23.4) | 192(25.2) | 157(11.7) |
| 2Z< BW≦3Z |  | 249(9.8) | 60(9.1) | 73(9.6) | 31(2.3) |
| 3Z< BW |  | 57(2.1) | 15(2.3) | 17(2.2) | 4(0.3) |
| BL,  Z scores, n(%) | | | | | |
| BL≦-3Z | 0.064  (0.000) | 21(0.8) | 8(1.2) | 4(0.5) | 73(5.4) |
| -3Z< BL≦-2Z |  | 171(6.8) | 58(8.8) | 43(5.6) | 126(9.4) |
| -2Z< BL≦-Z |  | 244(9.6) | 57(8.6) | 95(12.4) | 260(19.3) |
| -Z< BL≦Mean |  | 504(19.8) | 130(19.7) | 156(20.5) | 351(26.1) |
| Mean< BL≦Z |  | 691(27.2) | 178(27.0) | 194(25.5) | 321(23.8) |
| Z< BL≦2Z |  | 581(22.8) | 145(22.0) | 165(21.6) | 162(12.0) |
| 2Z< BL≦3Z |  | 263(10.3) | 66(10.0) | 86(11.3) | 40(3.0) |
| 3Z< BL |  | 69(2.7) | 18(2.7) | 20(2.6) | 14(1.0) |
| HC,  Z scores, n(%) | | | | | |
| HC≦-3Z | 0.098  (3.24E-08) | 13(0.5) | 3(0.5) | 4(0.5) | 85(6.3) |
| -3Z< HC≦-2Z |  | 113(4.4) | 38(5.8) | 32(4.2) | 109(8.1) |
| -2Z< HC≦-Z |  | 237(9.4) | 71(10.8) | 80(10.5) | 289(21.5) |
| -Z< HC≦Mean |  | 482(19.0) | 140(21.2) | 142(18.6) | 386(28.7) |
| Mean< HC≦Z |  | 809(31.8) | 206(31.2) | 236(30.9) | 295(21.9) |
| Z< HC≦2Z |  | 601(23.6) | 138(20.9) | 180(23.7) | 147(10.9) |
| 2Z< HC≦3Z |  | 245(9.6) | 50(7.6) | 78(10.2) | 32(2.4) |
| 3Z< HC |  | 44(1.7) | 14(2.0) | 11(1.4) | 3(0.2) |
| BSID-III Cognitive score, mean (SD) | 0.253  (0) | 95.9(12.7) | 92.9(14.1) | 95.9(12.6) | 97.8(12.6) |
| BSID-III Motor score, mean (SD) | 0.257  (0) | 92.5(15.1) | 88.96(16.1) | 92.6(14.9) | 94.0(14.5) |
| **Variables while regular follow up at 12 months CA** | | | | | |
| BW,  Z scores, n (%) | | | | | |
| BW≦-3Z | 0.113  (1.65E-10) | 7(0.2) | 0(0.0) | 3(0.4) | 39(2.9) |
| -3Z< BW≦-2Z |  | 78(3.1) | 42(6.3) | 17(2.2) | 131(9.8) |
| -2Z< BW≦-Z |  | 204(8.0) | 58(8.8) | 61(8.0) | 299(22.4) |
| -Z< BW≦Mean |  | 560(22.0) | 155(23.5) | 176(23.1) | 363(27.2) |
| Mean< BW≦Z |  | 869(34.2) | 206(31.3) | 266(34.9) | 332(24.9) |
| Z< BW≦2Z |  | 616(24.2) | 151(22.9) | 178(23.3) | 115(8.6) |
| 2Z< BW≦3Z |  | 183(7.2) | 40(6.0) | 53(7.0) | 23(1.7) |
| 3Z< BW |  | 27(1.1) | 8(1.2) | 9(1.1) | 10(0.7) |
| BH,  Z scores, n (%) | | | | | |
| BH≦-3Z | 0.064  (0.000) | 15(0.6) | 4(0.6) | 8(1.1) | 63(4.7) |
| -3Z< BH≦-2Z |  | 124(4.9) | 47(7.1) | 34(4.5) | 131(9.8) |
| -2Z< BH≦-Z |  | 284(11.1) | 89(13.5) | 96(12.6) | 299(22.4) |
| -Z< BH≦Mean |  | 501(19.7) | 128(19.4) | 152(19.9) | 363(27.2) |
| Mean< BH≦Z |  | 785(30.9) | 178(27.0) | 228(29.9) | 332(24.9) |
| Z< BH≦2Z |  | 566(22.2) | 146(22.1) | 159(20.8) | 115(8.6) |
| 2Z< BH≦3Z |  | 211(8.3) | 55(8.3) | 67(8.7) | 23(1.7) |
| 3Z< BH |  | 58(2.3) | 13(2.0) | 19(2.5) | 10(0.7) |
| HC,  Z scores, n (%) | | | | | |
| HC≦-3Z | 0.149  (0) | 15(0.6) | 3(0.5) | 4(0.5) | 74(5.5) |
| -3Z< HC≦-2Z |  | 96(3.8) | 51(7.7) | 21(2.8) | 115(8.5) |
| -2Z< HC≦-Z |  | 217(8.5) | 63(9.6) | 67(8.8) | 269(20.0) |
| -Z< HC≦Mean |  | 530(20.8) | 152(23.0) | 167(21.9) | 464(34.5) |
| Mean< HC≦Z |  | 822(32.3) | 194(29.4) | 239(31.3) | 273(20.3) |
| Z< HC≦2Z |  | 611(24.0) | 143(21.6) | 184(24.1) | 117(8.7) |
| 2Z< HC≦3Z |  | 218(8.6) | 45(6.8) | 73(9.6) | 30(2.2) |
| 3Z< HC |  | 35(1.4) | 9(1.4) | 8(1.0) | 4(0.3) |
| BSID-III Cognitive score, mean (SD) | 0.357  (0) | 98.7(12.0) | 95.6(14.2) | 98.8(11.6) | 99.0(12.6) |
| BSID-III Motor score, mean (SD) | 0.439  (0) | 92.0(13.4) | 87.9(16.4) | 92.4(12.9) | 92.3(13.5) |
| **CRegres: BSIDIII Cognitive score declines≧15 between 6 and 24 months CA** | | | | | |
|  | | No (%) |  | |  |
| characters |  | Original  cohort  (n=2544) | Train  (Balance)  (n=846) | Independent  Test  (n=764) | External cohort  (n=1347) |
| **Pregnant variables for time point before delivery** | | | | | |
| Maternal education, less than 12 years, n (%) | -0.067  (0.000) | 810(31.8) | 279(32.9) | 252(33.0) | 533(39.6) |
| Paternal education, less than 12 years, n (%) | -0.045  (0.011) | 847(33.3) | 296(34.9) | 261(34.2) | 532(39.5) |
| **Pregnant variables for time point before delivery** | | | | | |
| MgSO4, n (%) | 0.065  (0.000) | 776(30.5) | 229(27.1) | 246(32.2) | 702(52.1) |
| First child, n (%) | 0.046  (0.010) | 1508(59.2) | 503(59.5) | 446(58.4) | 807(59.9) |
| PROM, n (%) | -0.036  (0.040) | 890(34.9) | 311(36.8) | 270(35.4) | 441(32.7) |
| **Neonates’ variables for time point of admission** | | | | | |
| Male gender, n (%) | -0.036  (0.043) | 1295(50.9) | 421(49.7) | 423(55.4) | 716(53.2) |
| BW,  Z scores, n (%) | | | | | |
| BW≦-3Z | -0.071  (6.32E-05) | 24(0.9) | 8(0.9) | 6(0.8) | 75(5.6) |
| -3Z< BW≦-2Z |  | 69(2.7) | 11(1.3) | 20(2.6) | 89(6.6) |
| -2Z< BW≦-Z |  | 179(7.0) | 43(5.1) | 60(7.9) | 245(18.2) |
| -Z< BW≦Mean |  | 403(15.8) | 137(16.2) | 120(15.7) | 568(42.2) |
| Mean< BW≦Z |  | 1161(45.6) | 403(47.6) | 342(44.8) | 349(25.9) |
| Z< BW≦2Z |  | 665(26.2) | 229(27.1) | 201(26.3) | 20(1.4) |
| 2Z< BW≦3Z |  | 42(1.7) | 15(1.8) | 14(1.9) | 1(0.1) |
| 3Z< BW |  | 1(0.1) | 0(0.0) | 0(0.0) | 0(0.0) |
| HC,  Z scores, n (%) | | | | | |
| HC≦-3Z | -0.081  (5.62E-06) | 31(1.2) | 9(1.1) | 9(1.2) | 26(1.9) |
| -3Z< HC≦-2Z |  | 39(1.6) | 25(2.9) | 7(0.9) | 96(7.1) |
| -2Z< HC≦-Z |  | 163(6.4) | 67(7.9) | 59(7.7) | 349(25.9) |
| -Z< HC≦Mean |  | 687(27.0) | 218(25.8) | 197(25.8) | 564(41.9) |
| Mean< HC≦Z |  | 1105(43.4) | 370(43.8) | 335(43.9) | 272(20.2) |
| Z< HC≦2Z |  | 463(18.2) | 141(16.6) | 134(17.6) | 34(2.5) |
| 2Z< HC≦3Z |  | 49(1.9) | 15(1.8) | 21(2.8) | 4(0.3) |
| 3Z< HC |  | 7(0.3) | 1(0.1) | 1(0.1) | 0(0.0) |
| BL,  Z scores, n (%) | | | | | |
| BL≦-3Z | -0.057  (0.001) | 26(1.0) | 9(1.1) | 7(0.9) | 48(3.2) |
| -3Z< BL≦-2Z |  | 81(3.2) | 25(3.0) | 21(2.8) | 124(9.2) |
| -2Z< BL≦-Z |  | 258(10.1) | 67(7.9) | 84(11.0) | 402(30.2) |
| -Z< BL≦Mean |  | 669(26.3) | 218(25.7) | 206(27.0) | 537(39.9) |
| Mean< BL≦Z |  | 1031(40.5) | 370(43.7) | 310(40.6) | 190(26.5) |
| Z< BL≦2Z |  | 420(16.5) | 141(16.7) | 115(15.0) | 39(2.9) |
| 2Z< BL≦3Z |  | 50(2.0) | 15(1.8) | 15(2.0) | 2(0.1) |
| 3Z< BL |  | 9(0.4) | 1(0.1) | 5(0.7) | 0(0.0) |
| 1min Apgar score, (SD) | -0.069  (0.000) | 5.5(2.0) | 5.7(2.0) | 5.5(2.0) | 5.6(2.0) |
| 5min Apgar score, (SD) | -0.070  (7.77E-05) | 7.6(1.6) | 7.70(1.6) | 7.58(1.6) | 7.8(1.7) |
| SGA, n (%) | 0.079  (8.34E-06) | 468(18.4) | 130(15.4) | 164(21.5) | 219(16.3) |
| 1^st^ time Blood Sugar, mg/dl (SD) | -0.035  (0.049) | 75.0(34.0) | 76.2(35.4) | 76.4(35.3) | 73.8(34.7) |
| **Lab data variables during the hospitalization** | | | | | |
| Peak bilirubin level, mean (SD), mg/dl | 0.036  (0.042) | 9.0(3.0) | 9.0(2.8) | 9.1(2.9) | none |
| **Brain sonography variables during hospitalization** | | | | | |
| PVE, n (%) | 0.081  (4.46E-06) | 1136(44.6) | 374(44.2) | 347(45.4) | 573(42.5) |
| **Medication and treatment variables during the hospitalization** | | | | | |
| iNO, n (%) | 0.037  (0.036) | 140(5.5) | 43(5.1) | 49(6.4) | 55(4.1) |
| Ibuprofen, n (%) | -0.039  (0.028) | 743(29.2) | 239(28.3) | 245(32.1) | 440(32.7) |
| **Diagnosis variables during the hospitalization** | | | | | |
| BPD, n (%) | 0.036  (0.039) | 1161(45.6) | 361(42.7) | 362(47.4) | 695(51.6) |
| **Respiratory variables during the hospitalization** | | | | | |
| IPPV days, mean (SD), d | 0.057  (0.001) | 12.2(21.3) | 11.7(21.1) | 11.6(20.4) | 10.2(17.3) |
| Respiratory support days, mean (SD), d | 0.050  (0.005) | 58.4(39.3) | 56.9(36.2) | 59.8(40.3) | 60.3(36.2) |
| PMA without respiratory support, mean (SD), wk | 0.089  (4.80E-07) | 35.8(4.3) | 38.6(3.5) | 36.1(4.6) | 36.1(4.0) |
| **Discharge variables** | | | | | |
| NICU days, mean (SD), d | 0.096  (6.84E-08) | 54.9(37.2) | 53.11(41.3) | 55.18(34.3) | 57.7(35.6) |
| O2 supply after discharge, n (%) | 0.056  (0.001) | 345(13.6) | 119(14.1) | 87(11.4) | 234(17.4) |
| BW,  Z scores, n (%) | | | | | |
| BW≦-3Z | -0.040  (0.023) | 45(1.8) | 15(1.8) | 18(2.3) | 88(8.2) |
| -3Z< BW≦-2Z |  | 124(4.9) | 31(3.7) | 34(4.4) | 131(9.7) |
| -2Z< BW≦-Z |  | 270(10.6) | 79(9.3) | 87(11.4) | 295(21.9) |
| -Z< BW≦Mean |  | 753(29.6) | 244(28.8) | 228(29.9) | 489(36.3) |
| Mean< BW≦Z |  | 916(36.0) | 344(40.6) | 267(35.0) | 279(20.7) |
| Z< BW≦2Z |  | 401(15.7) | 125(14.9) | 118(15.5) | 63(4.7) |
| 2Z< BW≦3Z |  | 33(1.3) | 8(0.9) | 11(1.5) | 1(0.07) |
| 3Z< BW |  | 2(0.1) | 0(0.0) | 0(0.0) | 1(0.07) |
| PMA while discharge, mean (SD), wk | 0.1669  (0) | 38.9(4.0) | 39.6(4.9) | 39.0(4.2) | 39.4(4.4) |
| Hearing impairment, n (%) | 0.057  (0.001) | 132(5.2) | 44(5.2) | 39(5.1) | 87(6.5) |
| **Variables while regular follow up at 6 months CA** | | | | | |
| BW,  Z scores, n (%) | | | | | |
| BW≦-3Z | -0.040  (0.023) | 22 (0.9) | 5 (0.6) | 7 (0.9) | 55(4.1) |
| -3Z< BW≦-2Z |  | 115 (4.5) | 29 (3.4) | 24 (3.2) | 98(7.3) |
| -2Z< BW≦-Z |  | 216 (8.5) | 73 (8.6) | 71 (9.3) | 259 (19.2) |
| -Z< BW≦Mean |  | 484 (19.1) | 171 (20.2) | 162 (21.2) | 386(28.7) |
| Mean< BW≦Z |  | 752 (29.6) | 245 (29.0) | 217 (28.4) | 357 (26.5) |
| Z< BW≦2Z |  | 649 (25.5) | 220 (26.0) | 192 (25.2) | 157(11.7) |
| 2Z< BW≦3Z |  | 249 (9.8) | 90 (10.7) | 73 (9.6) | 31(2.3) |
| 3Z< BW |  | 57 (2.1) | 13 (1.5) | 17 (2.2) | 4 (0.3) |
| BSID-III Cognitive score, mean (SD) | -0.617  (0) | 95.9 (12.7) | 98.4(12.5) | 95.9 (12.6) | 97.8(12.6) |
| BSID-III Motor score, mean (SD) | -0.355  (0) | 92.5(15.1) | 97.8(15.2) | 92.6 (14.9) | 94.0(14.5) |

| **MRegres: BSIDIII Motor score declines≧15 between 6 and 24 months CA** | | | | | |
| --- | --- | --- | --- | --- | --- |
|  | | No (%) |  | | |
| characters |  | Original  cohort  (n=2544) | Train  (Balance)  (n=696) | Independent  Test  (n=763) | External cohort  (n=1347) |
| **Pregnant variables for time point before delivery** | | | | | |
| Antenatal steroid, n, (%) | -0.036  (0.041) | None:  507 (19.9)  One dose:  513 (20.1)  Two or more dose:  1524 (60.0) | None:  158 (22.7)  One dose:  128 (18.3)  Two or more dose:  410 (60.0) | None:  146 (19.2)  One dose:  156 (20.4)  Two or more dose:  461 (60.4) | None: 255(19.0)  One dose:  245(18.2)  Two or more dose: 845(62.8) |
| PROM, n (%) | -0.040  (0.023) | 890(34.9) | 268(38.5%) | 259(33.9%) | 441(32.7) |
| **Neonates’ variables for time point of admission** | | | | | |
| BW,  Z scores, n (%) | | | | | |
| BW≦-3Z | -0.112  (2.43E-10) | 24(0.9) | 4(0.6) | 6(0.8) | 75(5.6) |
| -3Z< BW≦-2Z |  | 69(2.7) | 17(2.4) | 20(2.6) | 89(6.6) |
| -2Z< BW≦-Z |  | 179(7.0) | 32(4.6) | 60(7.9) | 245(18.2) |
| -Z< BW≦Mean |  | 403(15.8) | 100(14.4) | 120(15.7) | 568(42.2) |
| Mean< BW≦Z |  | 1161(45.6) | 338(48.6) | 342(44.8) | 349(25.9) |
| Z< BW≦2Z |  | 665(26.2) | 195(28.0) | 201(26.3) | 20(1.4) |
| 2Z< BW≦3Z |  | 42(1.7) | 9(1.3) | 14(1.9) | 1(0.1) |
| 3Z< BW |  | 1(0.1) | 1(0.1) | 0(0.0) | 0(0.0) |
| BL,  Z scores, n (%) | | | | | |
| BL≦-3Z | -0.097  (5.06E-08) | 26(0.9) | 4(0.5) | 7(0.9) | 48(3.2) |
| -3Z< BL≦-2Z |  | 81(3.5) | 22(3.2) | 21(2.8) | 124(9.2) |
| -2Z< BL≦-Z |  | 258(10.0) | 68(9.8) | 84(11.0) | 402(30.2) |
| -Z< BL≦Mean |  | 669(26.3) | 164(23.6) | 206(27.0) | 537(39.9) |
| Mean< BL≦Z |  | 1031(40.5) | 303(43.5) | 310(40.6) | 190(26.5) |
| Z< BL≦2Z |  | 420(16.5) | 115(16.5) | 115(15.0) | 39(2.9) |
| 2Z< BL≦3Z |  | 50(1.9) | 16(2.3) | 15(2.0) | 2(0.1) |
| 3Z< BL |  | 9(0.4) | 4(0.6) | 5(0.7) | 0(0.0) |
| HC,  Z scores, n (%) | | | | | |
| HC≦-3Z | -0.110  (5.42E-10) | 31(1.2) | 5(0.7) | 9(1.2) | 26(1.9) |
| -3Z< HC≦-2Z |  | 39(1.6) | 10(1.4) | 7(0.9) | 96(7.1) |
| -2Z< HC≦-Z |  | 163(6.4) | 38(5.4) | 59(7.7) | 349(25.9) |
| -Z< HC≦Mean |  | 687(27.0) | 182(26.2) | 197(25.8) | 564(41.9) |
| Mean< HC≦Z |  | 1105(43.4) | 302(43.4) | 335(43.9) | 272(20.2) |
| Z< HC≦2Z |  | 463(18.2) | 142(20.4) | 134(17.6) | 34(2.5) |
| 2Z< HC≦3Z |  | 49(1.9) | 15(2.2) | 21(2.8) | 4(0.3) |
| 3Z< HC |  | 7(0.3) | 2(0.3) | 1(0.1) | 0(0.0) |
| 1min Apgar score, (SD) | -0.070  (7.68E-05) | 5.53(2.0) | 5.7(2.0) | 5.5(2.0) | 5.6(2.0) |
| 5min Apgar score, (SD) | -0.070  (7.20E-05) | 7.57(1.6) | 7.7(1.51) | 7.6(1.6) | 7.8(1.7) |
| SGA, n (%) | 0.118  (3.10E-11) | 474(18.6) | 102(14.7) | 146(19.1) | 219(16.3) |
| 1^st^ time Body temperature, °C (SD) | 0.036  (0.045) | 36.0(1.2) | 35.9(1.0) | 36.0(1.3) | 35.7(2.7) |
| 1^st^ time Blood Sugar, mg/dl (SD) | -0.063  (0.000) | 75.0(34.0) | 75.8(34.5) | 76.4(35.3) | 73.8(34.7) |
| BE in 1st time blood gas, mEq/dl SD | -0.042  (0.019) | -4.0(4.5) | -4.0(5.0) | -4.0(5.4) | 4.1(4.0) |
| **Medication and treatment variables during the hospitalization** | | | | | |
| Abdominal surgery, n, (%) | 0.046  (0.009) | 28(1.1) | 4(0.6) | 6(0.8) | 45(3.3) |
| Blood transfusion, n (%) | 0.047  (0.007) | 1917(75.4) | 515(73.9) | 589(77.2) | none |
| iNO, n (%) | 0.039  (0.027) | 140(5.5) | 30(4.3) | 38(4.9) | 55(4.1) |
| **Diagnosis variables during the hospitalization** | | | | | |
| BPD, n (%) | 0.084  (2.21E-06) | 1161(45.6) | 278(39.9) | 362(47.4) | 695(51.6) |
| Sepsis, n (%) | 0.042  (0.019) | 494(19.4) | 123(17.7) | 155(20.3) | 114(8.5) |
| **Respiratory variables during the hospitalization** | | | | | |
| High oxygenation supply, n (%) | 0.039  (0.030) | 1078(42.4) | 267(38.4) | 328(43.0) | 530(39.3) |
| IPPV days, mean (SD) , d | 0.117  (3.65E-11) | 12.2 (21.3) | 9.9(18.9) | 11.6(20.4) | 10.2(17.3) |
| Respiratory support days, mean (SD), d | 0.086  (1.13E-06) | 58.4(39.3) | 53.8(38.9) | 59.8(40.3) | 60.3(36.3) |
| PMA without respiratory support, mean (SD), wk | 0.132  (7.19E-14) | 35.8(4.3) | 35.4(4.2) | 36.1(4.6) | 36.1(3.9) |
| **Discharge variables** | | | | | |
| NICU days, mean (SD), d | 0.099  (2.68E-08) | 54.9(37.2) | 51.9(43.6) | 56.8(45.6) | 57.7(35.6) |
| O2 supply after discharge, n (%) | 0.035  (0.043) | 345(13.6) | 83(11.9) | 103(13.5) | 234(17.4) |
| PMA while discharge, mean (SD), wk | 0.167(0) | 38.9(4.0) | 38.5(3.9) | 39.0(4.2) | 39.4(4.4) |
| **Variables while regular follow up at 6 months CA** | | | | | |
| Caretaker | 0.036  (0.044) | Mother  1751 (68.8)  Grandparents  546(21.5)  Others  247(9.7) | Mother  470  (67.5)  Grandparents  162(23.3)  Others  64(9.2) | Mother  517  (67.8)  Grandparents  168(22.0)  Others  78(10.2) | Mother  1002  (74.4)  Grandparents  160(11.9)  Others  185(13.7) |
| BL,  Z scores, n (%) | | | | | |
| BL≦-3Z | -0.060  (0.000) | 21(0.8) | 8(1.2) | 4(0.5) | 73(5.4) |
| -3Z< BL≦-2Z |  | 171(6.8) | 44(6.3) | 43(5.6) | 126(9.4) |
| -2Z< BL≦-Z |  | 244(9.6) | 65(9.3) | 95(12.4) | 260(19.3) |
| -Z< BL≦Mean |  | 504(19.8) | 133(19.1) | 156(20.5) | 351(26.1) |
| Mean< BL≦Z |  | 691(27.2) | 187(26.9) | 194(25.5) | 321(23.8) |
| Z< BL≦2Z |  | 581(22.8) | 167(24.0) | 165(21.6) | 162(12.0) |
| 2Z< BL≦3Z |  | 263(10.3) | 72(10.3) | 86(11.3) | 40(3.0) |
| 3Z< BL |  | 69(2.7) | 20(2.9) | 20(2.6) | 14(1.0) |
| HC,  Z scores, n (%) | | | | | |
| HC≦-3Z | -0.080  (5.80E-06) | 13(0.5) | 2(0.2) | 4(0.5) | 85(6.3) |
| -3Z< HC≦-2Z |  | 113(4.4) | 31(4.5) | 32(4.2) | 109(8.1) |
| -2Z< HC≦-Z |  | 237(9.4) | 60(8.6) | 80(10.5) | 289(21.5) |
| -Z< HC≦Mean |  | 482(19.0) | 131(18.8) | 142(18.6) | 386(28.7) |
| Mean< HC≦Z |  | 809(31.8) | 230(33.1) | 236(30.9) | 295(21.9) |
| Z< HC≦2Z |  | 601(23.6) | 164(23.6) | 180(23.7) | 147(10.9) |
| 2Z< HC≦3Z |  | 245(9.6) | 66(9.5) | 78(10.2) | 32(2.4) |
| 3Z< HC |  | 44(1.7) | 12(1.7) | 11(1.4) | 3(0.2) |
| BW,  Z scores, n (%) | | | | | |
| BW≦-3Z | -0.094  (1.02E-07) | 22(0.9) | 6(0.9) | 7(0.9) | 55(4.1) |
| -3Z< BW≦-2Z |  | 115(4.5) | 21(3.0) | 24(3.2) | 98(7.3) |
| -2Z< BW≦-Z |  | 216(8.5) | 47(6.8) | 71(9.3) | 259(19.2) |
| -Z< BW≦Mean |  | 484(19.1) | 135(19.4) | 162(21.2) | 386(28.7) |
| Mean< BW≦Z |  | 752(29.6) | 218(31.3) | 217(28.4) | 357(26.5) |
| Z< BW≦2Z |  | 649(25.5) | 181(26.0) | 192(25.2) | 157(11.7) |
| 2Z< BW≦3Z |  | 249(9.8) | 69(9.9) | 73(9.6) | 31(2.3) |
| 3Z< BW |  | 57(2.1) | 19(2.7) | 17(2.2) | 4(0.3) |
| BSID-III Cognitive score, mean (SD) | -0.397  (0) | 95.9(12.7) | 98.4(12.5) | 95.9(12.6) | 97.8 (12.6) |
| BSID-III Motor score, mean (SD) | -0.689  (0) | 92.5(15.1) | 97.8(15.2) | 92.6(14.9) | 94.0 (14.5) |
|  | | | | | |
