# Supplementary material for: Developing a practical neurodevelopmental prediction model for targeting high-risk very preterm infants during visit after NICU: a retrospective national longitudinal cohort study
Source: BMC Med. 2024 Feb 16;22:68. doi: 10.1186/s12916-024-03286-2 (PMC10870669; doi:10.1186/s12916-024-03286-2)
Supplement: Supplementary file 3 — Additional file 3. Performance of EL-NDI and RF in independent test and external cohort. [file 12916_2024_3286_MOESM3_ESM.docx]

**Additional file 3. Performance of EL-NDI and RF in independent test and external cohort**

| **BSIDIII Cognitive score < 85 at 24 months CA** | | | | | |
| --- | --- | --- | --- | --- | --- |
| **EL-NDI** | **2010-2014**  **Multiple-center cohort**  **Independent test (n=763)** | | | **2016-2017**  **Multiple-center cohort**  **External test (n=1347)** | |
|  | Cognitive  (**CDelay**) | Motor  (**MDelay**) | | Cognitive  (**CDelay**) | Motor  (**MDelay**) |
| Accuracy | 77.6% | 75.4% | | 75.1% | 79.1% |
| AUC | 0.75 | 0.73 | | 0.78 | 0.82 |
| 95% CIs | 0.72-0.78 | 0.70-0.76 | | 0.75-0.82 | 0.79-0.85 |
| MCC | 0.277 | 0.317 | | 0.321 | 0.425 |
| Sensitivity | 50.0% | 56.7% | | 62.0% | 64.7% |
| Specificity | 82.5% | 79.6% | | 77.5% | 82.3% |
| PPV | 33.3% | 38.6% | | 34.5% | 48.4% |
| NPV | 90.4% | 89.0% | | 91.4% | 90.1% |
| LR ＋ | 2.86 | 2.78 | | 2.75 | 3.65 |
| LR － | 0.60 | 0.70 | | 0.47 | 0.48 |
| **BSIDIII Motor score declines≧15 between 6 and 24 months CA** | | | | | |
|  | Cognitive  (**CRegres**) | | Motor  (**MRegres**) | Cognitive  (**CRegres**) | Motor  (**MRegres**) |
| Accuracy | 73.0% | | 76.9% | 69.6% | 72.2% |
| AUC | 0.81 | | 0.83 | 0.76 | 0.79 |
| 95% CIs | 0.77~0.85 | | 0.80~0.86 | 0.74-0.78 | 0.78-0.80 |
| MCC | 0.412 | | 0.450 | 0.348 | 0.397 |
| Sensitivity | 74.6% | | 76.5% | 68.9% | 76.0% |
| Specificity | 72.6% | | 77.0% | 69.9% | 71.2% |
| PPV | 45.8% | | 44.7% | 44.6% | 41.8% |
| NPV | 90.2% | | 93.1% | 86.5% | 91.6% |
| LR ＋ | 2.73 | | 3.33 | 2.29 | 2.64 |
| LR － | 0.35 | | 0.24 | 0.44 | 0.40 |
|  | | | | | |

| **BSIDIII Cognitive score < 85 at 24 months CA** | | | | | |
| --- | --- | --- | --- | --- | --- |
| **RF** | **2010-2014**  **Multiple-center cohort**  **Independent test (n=763)** | | | **2016-2017**  **Multiple-center cohort**  **External test (n=1347)** | |
|  | Cognitive  (**CDelay**) | Motor  (**MDelay**) | | Cognitive  (**CDelay**) | Motor  (**MDelay**) |
| Accuracy | 76.7% | 73.0% | | 73.2% | 75.7% |
| AUC | 0.71 | 0.71 | | 0.78 | 0.82 |
| 95% CI | 0.65-0.76 | 0.66-0.77 | | 0.73-0.80 | 0.79-0.85 |
| MCC | 0.255 | 0.283 | | 0.310 | 0.415 |
| Sensitivity | 48.0% | 56.5% | | 64.5% | 71.6% |
| Specificity | 82.5% | 77.3% | | 74.8% | 76.8% |
| PPV | 58.2% | 69.9% | | 32.6% | 44.2% |
| NPV | 72.8% | 57.3% | | 91.8% | 91.3% |
| LR ＋ | 2.67 | 2.43 | | 2.56 | 3.08 |
| LR － | 0.63 | 0.57 | | 0.47 | 0.37 |
| **BSIDIII Motor score declines≧15 between 6 and 24 months CA** | | | | | |
|  | Cognitive  (**CRegres**) | | Motor  (**MRegres**) | Cognitive  (**CRegres**) | Motor  (**MRegres**) |
| Accuracy | 71.7% | | 81.8% | 61.6% | 75.6% |
| AUC | 0.78 | | 0.86 | 0.68 | 0.76 |
| 95% CI | 0.74-0.82 | | 0.83-0.90 | 0.64-0.71 | 0.73-0.79 |
| MCC | 0.383 | | 0.512 | 0.240 | 0.403 |
| Sensitivity | 72.3% | | 72.6% | 67.9% | 68.4% |
| Specificity | 72.6% | | 84.5% | 59.4% | 77.5% |
| PPV | 63.2% | | 88.9% | 36.9% | 45.3% |
| NPV | 44.8% | | 39.8% | 84.1% | 90.0% |
| LR ＋ | 2.57 | | 4.56 | 1.67 | 3.04 |
| LR － | 0.39 | | 0.32 | 0.54 | 0.41 |
|  | | | | | |
