# Supplementary material for: Developing a practical neurodevelopmental prediction model for targeting high-risk very preterm infants during visit after NICU: a retrospective national longitudinal cohort study
Source: BMC Med. 2024 Feb 16;22:68. doi: 10.1186/s12916-024-03286-2 (PMC10870669; doi:10.1186/s12916-024-03286-2)
Supplement: Supplementary file 4 — Additional file 4. Top five ranking variables chosen between RF with all features in methods and EL-NDI. Note: The main effect reveals the individual effect of a factor in prediction models. In calculating the influence of factors on the outcome, EL-NDI automatically computes the main effect values when the factor maximizes and minimizes its impact on the result, defining the absolute difference between these two values as MED. The most effective factor has the largest MED. [file 12916_2024_3286_MOESM4_ESM.docx]

| **Additional file 4 Top five ranking variables chosen between RF with all features in methods and EL-NDI** | | | | |
| --- | --- | --- | --- | --- |
|  | **RF** | Coefficient | **EL-NDI** | MED |
| **CDelay: BSIDIII Cognitive score < 85 at 24 months CA** | | | | |
| 1 | BSID-III Cognitive score  at CA 12  months | 0.111 | BSID-III  Motor score  at CA 12  months | 2.096 |
| 2 | BSID-III  Motor score at CA 12  months | 0.055 | BSID-III  Cognitive score  at CA 12  months | 1.087 |
| 3 | BSID-III Cognitive score at CA 6  months | 0.043 | Abdominal surgery | 0.908 |
| 4 | BSID-III  Motor score  at CA 6  months | 0.034 | IPPV days | 0.392 |
| 5 | First sodium bicarbonate  levels | 0.111 | First time  blood pH | 0.056 |
| **MDelay: BSIDIII Motor score < 85 at 24months CA** | | | | |
| 1 | BSID-III  Motor score  at CA 12  months | 0.671 | BSID-III  Motor score  at CA 12  months | 6.906 |
| 2 | BSID-III Cognitive score  at CA 12  months | 0.663 | BSID-III  Cognitive score  at CA 12  months | 3.312 |
| 3 | BSID-III Cognitive score  at CA 6  months | 0.513 | NICU days | 2.077 |
| 4 | BSID-III  Motor score  at CA 6  months | 0.391 | PMA  while discharge | 1.066 |
| 5 | NICU days | 0.373 | none |  |
| **CRegres: BSIDIII Cognitive score declines≧15 between 6 and 24 months CA** | | | | |
| 1 | BSID-III Cognitive scores  at CA6  months | 0.252 | BSID-III Cognitive scores  at CA 6  months | 16.816 |
| 2 | BSID-III  Motor score  at CA 6  months | 0.110 | BSID-III  Motor score  at CA 6  months | 1.345 |
| 3 | NICU  days | 0.042 | Maternal  MgSO4 use | 0.785 |
| 4 | First time  blood pH | 0.035 | Parental education ≦12 years | 0.785 |
| 5 | First time  blood sugar | 0.033 | none |  |
| **MRegres: BSIDIII Motor score declines≧15 between 6 and 24 months CA** | | | | |
| 1 | BSID-III  Motor score  at CA 6  months | 0.326 | BSID-III  Motor score  at CA 6  months | 15.103 |
| 2 | BSID-III Cognitive score at CA 6  months | 0.099 | Antenatal  steroid use | 0.168 |
| 3 | First blood pH levels | 0.039 | Head circumference  at admission | 0.056 |
| 4 | First base deficiency data | 0.032 | Prolonged rupture of membranes | 0.056 |
| 5 | NICU  days | 0.028 | none |  |
| Note: The main effect reveals the individual effect of a factor in prediction models. In calculating the influence of factors on the outcome, EL-NDI automatically computes the main effect values when the factor maximizes and minimizes its impact on the result, defining the absolute difference between these two values as MED. The most effective factor has the largest MED. | | | | |
